# Towards constructing a generalized structural 3D breathing human lung model based on experimental volumes, pressures, and strains

**DOI:** 10.1371/journal.pcbi.1012680

**Published:** 2025-01-13

**Authors:** Arif Badrou, Crystal A. Mariano, Gustavo O. Ramirez, Matthew Shankel, Nuno Rebelo, Mona Eskandari

**Affiliations:** 1 Department of Mechanical Engineering, University of California Riverside, Riverside, California, United States of America; 2 Nuno Rebelo Associates, LLC, Fremont, California, United States of America; 3 BREATHE Center, School of Medicine, University of California Riverside, Riverside, California, United States of America; 4 Department of Bioengineering, University of California Riverside, Riverside, California, United States of America; University of Tennessee Health Science Center College of Medicine Memphis, UNITED STATES OF AMERICA

## Abstract

Respiratory diseases represent a significant healthcare burden, as evidenced by the devastating impact of COVID-19. Biophysical models offer the possibility to anticipate system behavior and provide insights into physiological functions, advancements which are comparatively and notably nascent when it comes to pulmonary mechanics research. In this context, an Inverse Finite Element Analysis (IFEA) pipeline is developed to construct the first continuously ventilated three-dimensional structurally representative pulmonary model informed by both organ- and tissue-level breathing experiments from a cadaveric human lung. Here we construct a generalizable computational framework directly validated by pressure, volume, and strain measurements using a novel inflating apparatus interfaced with adapted, lung-specific, digital image correlation techniques. The parenchyma, pleura, and airways are represented with a poroelastic formulation to simulate pressure flows within the lung lobes, calibrating the model’s material properties with the global pressure-volume response and local tissue deformations strains. The optimization yielded the following shear moduli: parenchyma (2.8 kPa), airways (0.2 kPa), and pleura (1.7 Pa). The proposed complex multi-material model with multi-experimental inputs was successfully developed using human lung data, and reproduced the shape of the inflating pressure-volume curve and strain distribution values associated with pulmonary deformation. This advancement marks a significant step towards creating a generalizable human lung model for broad applications across animal models, such as porcine, mouse, and rat lungs to reproduce pathological states and improve performance investigations regarding medical therapeutics and intervention.

## Author summary

The lungs are susceptible to harmful diseases that can impact respiratory function, resulting in approximately 4 million deaths annually. Addressing these challenges is imperative and can benefit from predictive models to enhance our understanding of lung physiology and optimize interventions and therapies. However, the complexity of the lung structure and limited experimental investigations hinder the development of accurate models. To address this gap, we designed an experimental-computational pipeline, representing the continuous inflation associated with the breathing cycle along with global and local pressures, volumes, and strains experimental measures collected using a unique apparatus merged with digital image correlation. The computational model is calibrated using a cadaveric human lung donated from a transplant site. Key features of lung inflation are captured at both the organ and tissue level, bringing us a major step closer to clinically applicable models to understand various forms of pathology.

## Introduction

Despite recent advancements in pulmonary medicine, respiratory diseases continue to pose a significant healthcare challenge [1,2]. The number of sufferers from chronic respiratory diseases is estimated to have increased by 39% between 1990 and 2017 [3]. The consequences are profound, with over 4 million deaths annually worldwide [4], and an estimated cost of more than $170 billion in 2016 for the United States alone [5]. The emergence of new respiratory diseases such as COVID-19, combined with an increase in cases of COPD and allergy-induced respiratory diseases due to the impact of a changing climate, further exacerbates the situation [6]. Still, research on lung biomechanics remains in its early stages and our understanding is comparatively decades behind other medical fields such as cardiovascular, neurology, or oncology [7–9]. This nascent landscape coupled with growing pulmonary healthcare needs emphasizes the urgency of further investigations to understand the mechanics of breathing in order to improve the diagnosis and treatment of lung diseases.

Numerical simulations and predictive human lung models play a crucial role in respiratory medicine by empowering researchers to evaluate physiological theories and predict treatment efficacy, leading to more effective therapies without resorting to costly and time-intensive studies [10,11]. These models can help researchers understand lung mechanics by illustrating how the lung deforms and strains under varying amounts of air pressure and volume, which is essential for assessing lung function and health [12]. Such simulations and measures of tissue stretch may also provide valuable assistance in guiding the use of mechanical ventilation, helping to avoid ventilator-induced injuries [13]. Predictive models may also support the monitoring of disease progression and assessing structural changes longitudinally [14–16]. Augmenting such proof of concept studies with more complex fluid-solid interactions can eventually further enhance our ability to model drug delivery methods or complex surgical operations, such as lung resections or other invasive techniques [17,18], which typically alter the mechanics of breathing. Computational models are posed to contribute significantly to informing our understanding of disease progression and the effectiveness of medical interventions.

However, to date, the development of lung models has mostly centered on airflow within the bronchi to investigate particle deposition [18–21], gas exchange [22], and virus spread, as demonstrated in recent models of the transmission of SARS-CoV-2 infection [23,24]; these investigations predominantly utilize idealized geometry and presume airflow within a rigid network of airways.

More recent studies have aimed to construct solid mechanics (structural) models of the lungs. In Werner et al. [25] and Hasani et al. [26], a model of lung deformation was developed, but the material properties were assumed to be linear elastic and isotropic, (considered simplistic since the lungs exhibit highly non-linear, anisotropic, viscoelastic, and heterogeneous characteristics [27–30]). Li et al. utilized a varying Young’s modulus across the lung to account for inhomogeneity [31]. Al-Mayah et al. employed sophisticated material laws such as hyperelasticity in their study to develop a human lung model where contact was represented between the lungs and the chest wall [32]. These separate studies are similar in that they rely on evaluation methods based on landmark points, due to the absence of a universal standard validation approach. Furthermore, validation is typically conducted at specific, discrete stages (e.g. end-point inhalation) due to the lack of continuous experimental data.

In a comprehensive overview of pulmonary computational models [33], Neelakantan et al. notes recent emphasis on models integrating airflow and lung tissue response to comprehensively capture the intricate features of the respiratory system. Collectively, these endeavors highlight the ongoing efforts to enhance the realism and accuracy of pulmonary models that encapsulate both airflow and structure in understanding the complexities of the respiratory system. Studies have explored the use of Fluid-Structure Interaction (FSI) techniques, but these approaches are often deemed prohibitively expensive [34,35] and undermine future translational goals for eventual clinical use to enhance diagnosis and intervention. A few studies underscore the significance of adopting a poroelastic formulation [36–38] and acknowledge its potential to represent the air-tissue interaction between alveolar pressure and tissue deformation inherent to the porous state of the lung [39], but they face experimental validation limitations.

Recently, Maghsoudi-Ganjeh et al. [40] developed an Inverse Finite Element Analysis (IFEA) framework for the construction of a material model of a porcine lung specimen, which was validated with continuous experimental data offered by novel Digital Image Correlation (DIC). However, while this study considered the anisotropy and heterogeneous features absent in prior works, it was limited to a reduced-order surface representation of a pig lung, did not encompass the coupled organ volume and pressure global response, and excluded the role of airway, parenchyma, and pleura lung constituents.

This current study notably leverages the advancements offered by the aforementioned IFEA pipeline and the benefits afforded by the poroelastic formulation to develop the first continuously ventilated 3D structurally-representative pulmonary breathing model informed by both organ- and tissue-level experiments from a cadaveric human lung. This study is uniquely informed by experimental pressures, volumes, and strains obtained through a custom-designed electromechanical pressure-volume (PV) ventilation system in conjunction with previously established and validated DIC methods [37–39]. It utilizes continuous experimental data for validation, aided by additional features ensuring its accuracy and dynamic behavior in relation to real data. Incorporations of recent advanced methods to represent the lungs (e.g. poroelastic representation) coupled with the novel specific methods and data collections introduced here (e.g. airway structure utilization, global to local experimental measures for calibration, etc.) drives us to define the scope of this work as a proof of concept pipeline validated by a human lung case study. This approach then lays the foundation to next expand the performance evaluation of this framework to multitudes of human lungs for statistical considerations and assessments of pathological states and to extending to animal lung mechanics.

This study is structured as follows: first, we present a concise description of the PV ventilation system and unveil key characteristics related to human lung inflation, both globally through pressures and volumes, and locally with tissue deformation. These features are used to build the finite element (FE) human lung inflation model and to calibrate it using a IFEA pipeline. This framework is then illustrated using a cadaveric human lung, where we demonstrate robust alignment between model results and continuous experimental pressure, volume, and strain measurements for the first time. Finally, we provide a discussion highlighting the advantages and restrictions to the model and propose future directions.

## Material and methods

The primary objective of this study was to provide a proof of concept for a FE model and the calibration methods, where the material behavior is optimized to accurately produce a generic human lung mechanics model as defined globally by the typical S-shaped PV curve with two inflection points [41,42], and as defined locally by the non-uniformly distributed strains across the lobes [13,43], and complex deformation patterns [40,42]. As such, here we describe the experimental tests which were used to inform and validate the model, along with the generic human commercial geometry which was used to investigate the generalizability of this approach. We further specify the material properties of each lung constituent (parenchyma, airways, and pleura) as either offered by experiments from our lab or as optimized values throughout the IFEA procedure.

### Ethics statement

Anonymized human cadaveric lungs from Donor Network West (San Ramon, California) and were approved for IRB exemption given the study included de-identified postmortem human lung samples (University of California Riverside Office of Research Integrity and/or Institutional Board Review HS #20–180).

### Experimental investigations: Custom-designed PV ventilation system & DIC interface

**Fig 1:**
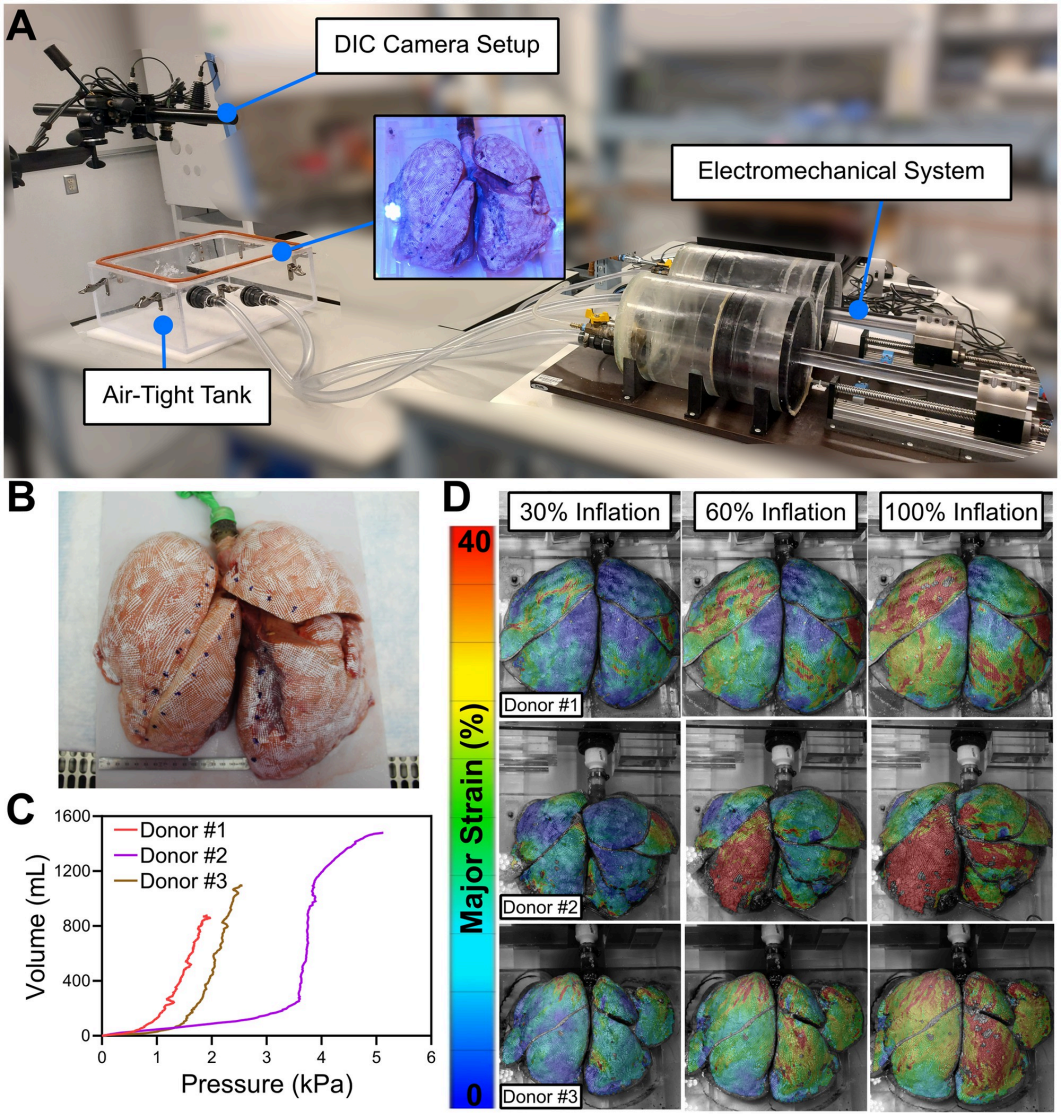
PV ventilation system coupled to DIC. **(A)** A unique global PV to local DIC interfaced system illustrates piston-actuated ventilation and overhead cameras capturing bulk pressures and volumes as associated with regional tissue strains. **(B)** Visualization of the speckling used to track the displacements and strains for DIC. **(C)** Various PV inflation trajectories continuously measured are exemplified for three donor lungs. **(D)** Inflation stages at 30%, 60%, and 100% of maximum pressure display non-trivial strain patterns.

Experimental data used to inform this study’s model utilized a customized ventilation system with the capability of recording continuous PV curves throughout inflation (Fig 1); the unique and extensively validated PV system has been previously described [41,44,45]. Briefly, the setup included two pistons, controlling volume and measuring pressure, to induce artificial ventilation (positive pressure applied to trachea, i.e., mechanical ventilation), and while also simultaneously measuring the actual volume change to the lung in real-time without post-calculations (Fig 1A). The human lungs were positioned atop a platform in a transparent tank, displaying the anatomical three lobes on the right side and two lobes on the left (Fig 1B). A 1X phosphate-buffered saline (PBS) solution was added to the platform to minimize friction between the lungs and the tank during inflation motion [13,43,46]. The trachea was connected to the piston system via a rigid plastic tube. The surface of each lung was speckled to associate the global response of the PV curve to the local behavior provided by the DIC technique using high-resolution, high-speed camera system to track displacements and strains, as extensively previously established [13,42,44,47,48]. As the constituents of the lungs are interconnected [49,50], alveolar expansion during breathing directly affects the pleura’s strain, supporting the use of experimental surface strain data to gain insights into the lung’s constituents in the IFEA.

PV curves generally exhibit two inflection points with a linear portion in between, maintaining a consistent shape (Fig 1C). Throughout inflation, concentrated strains were observed indicating uneven pressure distribution across the lungs, particularly in the initial stages (Fig 1D). This suggested regional expansion patterns, particularly that each lung lobe may exhibit a varying response. As inflation progressed, complex strain contours and lobar sliding were observed.

### FE model construction

#### Geometry and mesh of the parenchyma, airways, and pleura constituents

**Fig 2:**
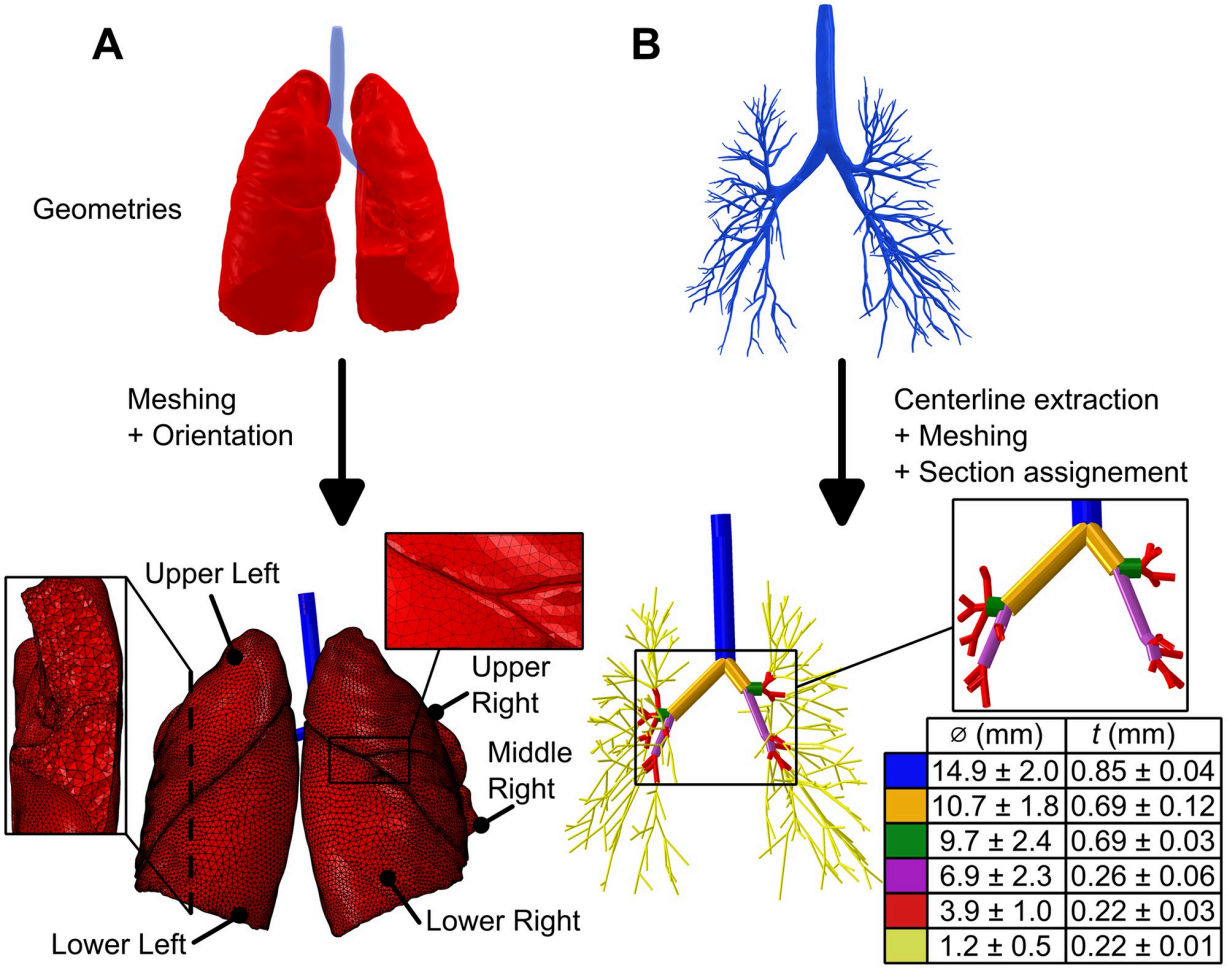
FEA geometry and mesh preparation. **(A)** The commercial geometry was meshed and oriented to reproduce the positioning of the specimen during inflation within the apparatus. The mesh was generated using HyperMesh with refinement based on curvature [51]. On the left, a perspective cut shows the utilization of smaller elements on the edges, while larger elements fill the bulk of the lungs. The refinement aided in modeling the contact between the lobes. **(B)** Airways from the commercial geometry up to the 10^th^ generation were used to extract centerlines and define the geometry.

A commercially available lung geometry (Zygote Media Group, Inc., USA) was employed to build the generic FE model in Abaqus (Dassault Systemes Simulia Corp., France). This geometry encompassed intricate details, including a volumetrically detailed parenchyma morphology with distinct lobes and fissures and the airway network (Fig 2A)

The main features of the lungs, including the airways, parenchyma, and pleura were represented in the FE model. For the 3D airways, the centerlines were extracted for the 10 generations available in the geometry, and the thicknesses and diameters were measured. Once these values were confirmed to be comparable to established values in the literature [52], 1266 beam elements were utilized where the assigned pipe section average geometries varied as listed (Fig 2B).

The pleura layer, which encompasses the soft spongy innards of the lungs was meshed using HyperMesh (Altair Engineering Inc., USA) with 50,719 linear triangular membrane elements (Fig 2A). Indeed, we assumed that the pleura does not exhibit any bending stiffness or out-of-plane stress, in line with the literature on thin and soft biological tissues [53,54]. The option to refine the mesh based on surface deviation was applied [51]. The parenchyma was meshed with 331,996 linear tetrahedral elements. The chosen number of elements was considered sufficient based on analyses of mesh convergence, to accurately model contact, and to represent necessary details of model geometric features for experimental validation, all while balancing the simulation cost.

### Material law for parenchyma, airways, and pleura components

The parenchyma was modeled with a poro-hyperlastic material law [55,56], where the strain density energy function *U*_*hyper*_ is as follows:

$$U_{hyper}=\;\frac{2\mu }{\alpha^{2}}\left[\lambda_{1}^{\alpha }+\lambda_{2}^{\alpha }+\lambda_{3}^{\alpha }-3+\frac{1}{\beta }\left(\text{det}{\left(\text{F}\right)}^{-\alpha \beta }-1\right)\right]$$ (1)

where *μ* is the initial shear modulus, *α* a coefficient, F the deformation gradient with a coupling between the volumetric and the deviatoric contributions [55], *λ*_*i*_ are the principal stretches such that det(F) = *λ*_1_*λ*_2_*λ*_3_, and *β* is linked to the Poisson’s ratio *ν* such that $\beta =\frac{\nu }{1-2\nu }$.

The hyperfoam law was coupled with permeability *k* in a poroelastic formulation to represent air diffusion in the lungs, relating air flow to gradients in pressure using a Forchheimer’s law [55]. Supporting Information (S2 Appendix) is available to provide additional context and explanation of the poroelastic formulation. As each lobe may respond differently to air flow [57,58], as observed in our experimental investigations, constant permeabilities were individually assigned to each of the five lobes (upper left, lower left, upper right, middle right and lower right; Fig 2A), resulting in five distinct parameters. A Neo-Hookean material law was used to model the airways defined by the strain energy potential *U*_*NH*_ such that:

$$U_{NH}=C_{10}\left(\bar{I_{1}}-3\right)+\frac{1}{D}{\left(\text{det}\left(\text{F}\right)-1\right)}^{2}$$ (2)

where *C*_10_ and *D* are material parameters and $\bar{I_{1}}$ is the first deviatoric strain invariant.

**Fig 3:**
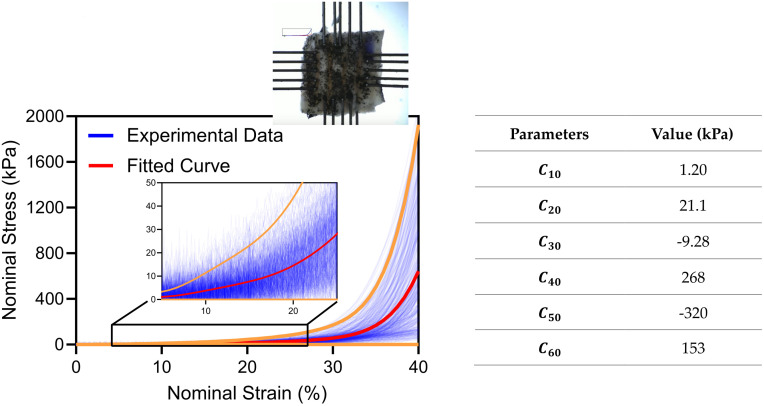
Experimental nominal stress-strain curves from the biaxial measurements on pleura samples used to inform the FE model. A polynomial curve was fit to all the data resulting in an averaged response (red) which generated material parameters using the Abaqus evaluation tool [55] for a reduced polynomial (Eq 3). The orange curves represent limiting cases where λ = 3 and λ = 1e^−6^ as defined in Table 1.

Unlike the parenchyma and airways, the material properties for the human pleura are unknown, hindering our evaluation of whether the FE model calibration would yield practical results. As such, we used experimentally tested specimens [59] to inform the simulations using 5 healthy (viable for transplant) and 4 non-healthy (e.g. smoker) lungs (age 17–63, 4 males and 5 females; IRB approved exemption, HS #20–180, Donor Network West, San Ramon, California). These samples were subject to equi-biaxial tensile testing (Cellscale Biomaterial Testing, Waterloo, Canada), as we have previously established [60–62], where 12 square samples of 1 cm x 1 cm area were extracted within 72 hours after the donor’s death, totaling 108 samples. We measured and assigned a thickness of 0.06 mm to the membrane elements representing the pleura, as corroborated by various studies [63,64]. An averaged curve was fitted to the stress-strain curves from all experiments to represent the wide variation in material properties, and this curve was further adjusted with a reduced polynomial hyperelastic law using Abaqus’ evaluation tool [55] and assuming incompressibility [53,65]. The strain energy potential of a reduced polynomial *U*_*RP*_ is presented in Eq 3 and the material properties corresponding to the shown biaxial response are shown in Fig 3. We checked that the obtained material is convex with respect to the Green-Lagrange strain tensor (S1 Appendix).


$$U_{RP}=\;\sum_{i=1}^{n}C_{i0}{\left(\bar{I_{1}}-3\right)}^{i}$$ (3)

### Boundary conditions and representation of experimental conditions

Contact was modeled between the lungs and the plate, as well as for lobar sliding, found recently to be an key player in lung deformation [66,67]. Contact was implemented using the general contact method with a penalty formulation for the normal behavior [55]. No friction is considered for the tangential behavior since smooth sliding of the lungs during this testing method has been previously confirmed [44]. As the model aimed to replicate the experimental inflation test in the tank, its orientation needed to match the physical positioning of the specimen on the platform (placed with the medial surface on the plate). A preliminary analysis was conducted to both rotate the lobes such that their placement in the tank was well represented, and to capture intricate details of the lobes pressing at the outward extremities of the specimens.

**Fig 4:**
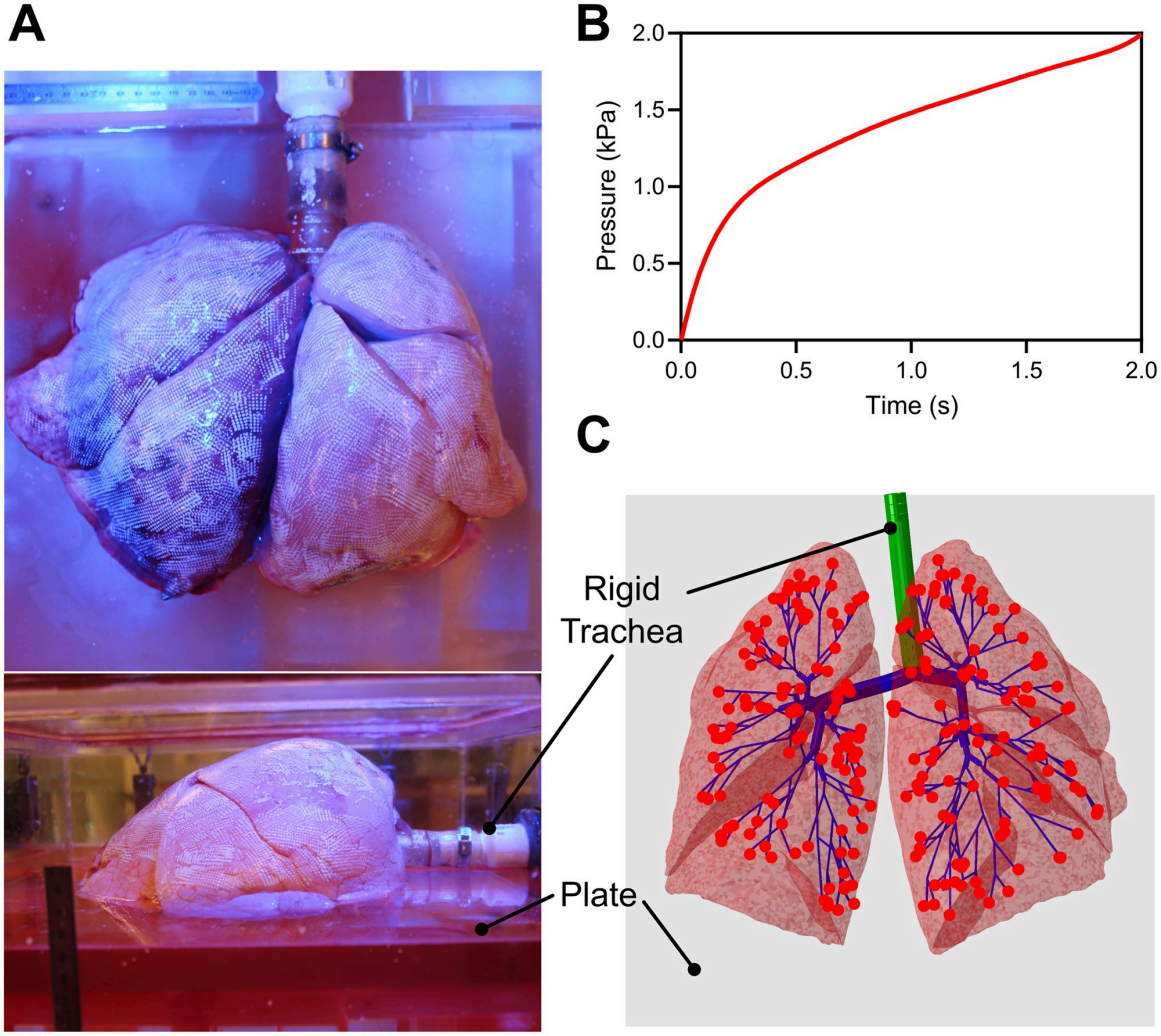
Boundary conditions. **(A)** Human lungs positioned medial face-down on the plate in the air-tight tank are inflated according to the experimentally recorded pressure evolution shown in **(B)**. The pressure values are increased at each of the 220 parenchymal nodes (red) corresponding to the end of the airway branches **(C)**, which then diffuse throughout the rest of the tissue. The trachea (green) is constrained, and the deformable airways (blue) are embedded into the solid elements of the lungs.

In the model, the airways were embedded in the parenchyma, similar to physiological conditions and are free to move [68,69]. The trachea was constrained to represent the rigid piping in the tank to which it was experimentally attached (Fig 4A), which was well supported by the absence of observed tracheal displacements. Increasing measured pressure values (Fig 4B), was applied directly to the parenchymal nodes near the ends of the airways (see Fig 4C) in a coupled transient stress-fluid analysis, employing a poroelastic formulation in a quasi-static manner [55]. A Python routine (Python Software Foundation, USA) was utilized to identify the closest parenchymal node for each node at the end of the branches. We chose to incorporate pressure directly on the nodes instead of modeling fluid flow through pipe elements as it was challenging to ensure proper connectivity between the fluid elements and the parenchyma [55,70], and to validate the pressure drop in the absence of literature (finding only that the pressure drop was minimal through the branches [80], which supported our alternative approach).

### IFEA calibration

#### Parameters and general IFEA process

To demonstrate FE model proof of concept, the material model was calibrated to achieve two main global (PV) and local (DIC) defining goals: the resulting mechanics should be well represented by (i) the S-shaped PV curve with lower and upper inflection points, and (ii) strains must be non-uniformly distributed with varying inflation patterns across the lobes (Fig 1). The generic human lung geometry was informed by one experimentally tested cadaveric human lung. Considering the rarity of receiving a donated functioning healthy human lung which is unpunctured and capable of inflation, the specific lung used here to conduct IFEA was obtained from a 34-year-old female brain-dead donor (IRB approved exemption #HS 20–180, Donor Network West, San Ramon, California), which was rejected from transplant and slated for research purposes from the onset due to poor pulmonary function (Fig 1D, Donor #1).

**Fig 5:**
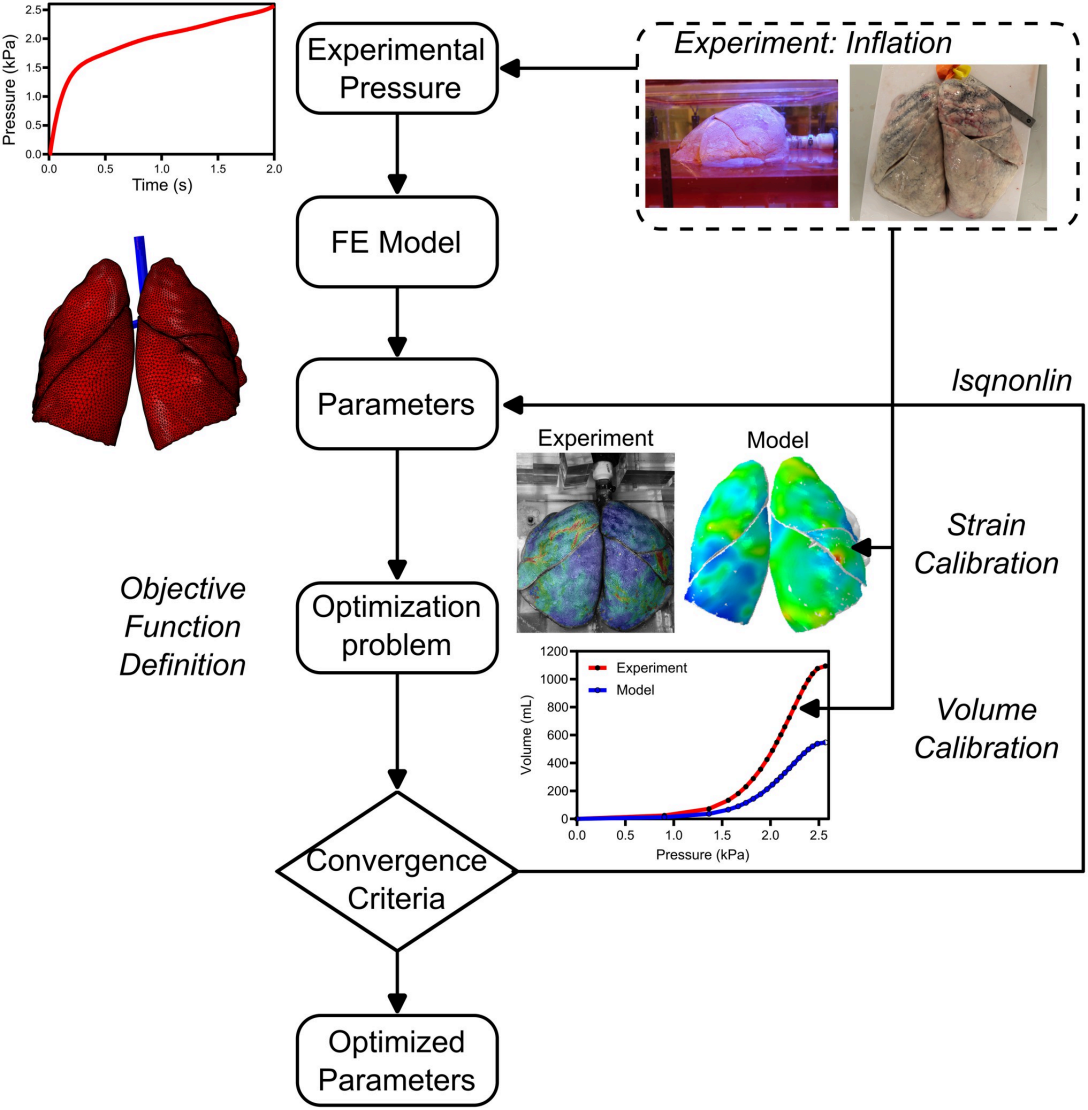
General IFEA optimization process used to model human lung mechanics. Experimental pressure values during inflation serve as the input, where nine model parameters were calibrated to minimize the difference between output global lung volume and local strain experiments.

The ventilation system can operate at various rates, but for this study, we concentrated on the physiological breathing rate of 15 breaths per minute as previously referenced [71,72], at an applied volume of 1090 mL, resulting in an actual lung volume of 934 mL and maximum pressure of 2.1 kPa The first step of the general IFEA procedure, defining the parameters in need of calibration, is illustrated in Fig 5.

For the parenchyma, the parameters *μ*, *α* and *ν* (Eq 1) were used to represent its behavior.

In this preliminary study, the assumption of incompressibility for the airways was made based on prior research findings [73–75]. Therefore, the material parameter *D* in Eq 2 was set to 0 and the only parameter was *C*_10_.

For the pleura, coefficients of a reduced polynomial model were derived through fitting the biaxial stress-strain curves, as previously mentioned. These obtained parameters are considered nonspecific due to observing widely varying pleura behavior mechanical response (seen in Fig 3 and previously reported [59]). To account for this, we introduced an additional parameter *λ* which serves as a coefficient applied to each parameter influencing the mechanical response of the pleura in Eq 3, such that the new material parameter $C_{i0}^{*}$ was defined as follows $C_{i0}^{*}={\lambda \times C}_{i0}$. This parameter *λ* enabled the adaptation of the pleural fit to specific lung characteristics while preserving the bilinear shape of the stress-strain curve. This approach maintains the bilinear aspect of the curve, as observed in instances where the pleura aids in restricting extension. We initially explored the direct use of the curve in the optimization process to incorporate eight parameters (instead of nine) and used the pleura’s material properties from the experimental data, but were met with challenges; beyond 40% strain, the pleura’s exponentially increasing stiffness made it inextensible. Moreover, preliminary biaxial tests revealed significant region-dependency [59], albeit not necessarily lobe dependent to justify varying the permeability.

Specified ranges for the nine parameters to be calibrated are listed in Table 1, and were defined through preliminary analyses, including a sensitivity analysis to evaluate their effects on the model (S3 Appendix), as well as insights from previous studies [30,76]. It was confirmed that values beyond these limits yielded unrealistic deformations or convergence difficulties.

**Table 1:** Parameters used in the optimization process and their boundaries. The parameters k_UL,LL,UR,MR,LR_ refer to the permeabilities for the five lobes as illustrated in Fig 4A. μ, α and ν are the material properties defined in Eq 1. The airways are characterized by the stiffness C_10_ from Eq 2, and the coefficient λ drives the mechanical behavior of the pleura based on Eq 3.

Lung Component	Parameters	Lower Value	Upper Value
Parenchyma	***μ* (kPa)**	0.1	100
	***α* (−)**	1.0e^−6^	10.0
	***ν* (−)**	0.0	0.5
	***k***_***UL*,*LL*,*UR*,*MR*,*LR***_ **(mm**^**2**^**.s**^**-1**^**)**	[0.1; 0.1; 0.1; 0.1; 0.1] × 10^−3^	[100; 100; 100; 100; 100] × 10^−3^
Airways	***C***_**10**_ **(kPa)**	0.1	100
Pleura	***λ* (−)**	1.0e^−6^	3

### Global calibration: Lung volume

To assess the similarity between the experiments and the model, experimental metrics and data goal definitions are needed to evaluate model calibration results. Here, we utilized recorded pressures (Fig 4B) as an input for the model and tracked the actual lung volume directly measured by the system, which is distinct from the applied volume of the system [41] and serves as the sum of the elementary volumes within the model [55].

The initial volume was set to zero for both experimental and model inflations, and only the added lung volumes were assessed. To accurately capture the shape of the PV curve, 20 points, representing pressure/volume pairs during inflation, were considered over the 2 s inflation, which corresponded to the 15 breaths per minute inflation rate.

The geometry of the lungs may vary, as shown in Fig 1D with various example lung cadavers; therefore, achieving identical volumes could result in different strains depending on the lung size. Therefore, to ensure meaningful data comparison, the entire generic model’s mesh was scaled to match the initial volume of the experimental lung. We define the initial volume $V_{air}^{0}$ as the air volume of the experimental lungs before inflation (when deflated) as follows:

$$V_{air}^{0}=V_{total}-V_{tissue}$$

where *V*_*total*_ is the total volume of the experimental lungs, measured using a submersion test [13]. *V*_*tissue*_ is the volume of the lung tissue, computed as follows:

$$V_{tissue}=\frac{m_{lung}}{\rho_{lung}}$$

where *m*_*lung*_ = 818 g is the weight of the donated lung and *ρ*_*lung*_ = 1.0 g/cm^3^ is the reported density of the deflated human lung tissue [77]. After the submersion test, we measured *V*_*total*_ = 2430 mL that led to $V_{air}^{0}=1612\;\text{mL}$, where $V_{air}^{0}$ represented the initial volume of air in the lungs.

As such, we define $V_{num}^{0}$ as the new initial volume of the model (the volume to match), and we scaled the mesh of the entire model so that $V_{num}^{0}=V_{total}$ with an initial porosity of $\frac{V_{air}^{0}}{V_{total}}=66\%$ assuming a fully saturated medium. Using this robust scaling method as directly informed by experiments, we compared the experiment and the model during inflation with the same initial volume of air.

### Lobe strains and local calibration

The non-identical geometries of the donated human lung and generic commercial lung required meaningful metrics for comparisons. The average and standard deviation for major strains (nominal principal strains) of each lung lobe provided regional and variational assessment to analyze potential experimental and computational discrepancies throughout the inflation stage [78]. The average strain and standard deviation values were extracted for each of the five lobes at 30%, 60%, and 100% of maximum pressure, resulting in 30 points of comparison. Consistent definition of the same visible contours of the lobes for both the computational model and experimental validation posed a challenge, since experimentally, the cameras have a limited view of the lobes which may vary from one lung to another and are manually defined. However, the applied methods used to define these view limits were replicated for the computational model, specifically contouring the lobes with a top view, just as was performed in experimental definitions.

### Objective function and optimization

To measure the similarity between the PV data and the strains, MATLAB’s *lsqnonlin* function (MathWorks Inc., USA) was utilized to minimize the objective function *φ*, and was defined as follows:

$${\text{min}}_{parameters}\varphi =\left\{\begin{array}{c} \sum_{i=1}^{20}\frac{\left|V_{exp,i}-V_{num,i}\right|}{\delta_{V}}\\ \sum_{i=1}^{15}\frac{\left|{\bar{\text{x}}}_{exp,i}-{\bar{\text{x}}}_{num,i}\right|}{\delta_{\bar{\text{x}}}}\\ \sum_{i=1}^{15}\frac{\left|\sigma_{exp,i}-\sigma_{num,i}\right|}{\delta_{\sigma }} \end{array}\right.$$ (4)

where the first component aimed to minimize the lung volume difference between the experimental volumes *V*_*exp*_ and the numerical volumes described by *V*_*num*_. The second component was used to match the average $\bar{\text{x}}$ major strains, and the third component sought to match the variability via the standard deviation *σ* of the major strain. As these three components are not in the same order of magnitude, the numerator was divided by the standard deviation of all the different experimental values for the volumes (*δ*_*V*_), for the average strain ($\delta_{\bar{\text{x}}}$), and for the strain deviations (*δ*_*σ*_) in order to normalize the objective function [79].

The trust-region-reflective algorithm was employed with a forward difference scheme to compute the derivative of the objective function [80]. Given that the general problem may result in multiple local minima, the *MultiStart* option was utilized with three starting points to explore a global minimum [81]. The parameter ranges defined through preliminary analyses also aided in narrowing down the search space. The search for a local minimum was terminated based on convergence criteria; that is, if any of the following conditions were met:

- *Function tolerance*: for iteration *i*, the objective function *φ*, and a set of parameters *x*_*i*_, if |*φ*(*x*_*i*_) − *φ*(*x*_*i*+1_)| < 1e^−6^ (1 + |*φ*(*x*_*i*_)|).
- *Maximum of function evaluation*: if the number of function evaluation exceeds 100 × number of parameters = 900.
- *Maximum of iterations*: if the number of iterations exceeds 400.
- *Optimality tolerance*: if we consider the first-order optimality measure, the solver will stop if max_*i*_ |*v*_*i*_ * *g*_*i*_| < 1e^−6^. *g*_*i*_ is the *i*th component of the gradient. For a current point *x*, the expression of *v*_*i*_ is defined such that:
vi=xi-biifthenegativegradientpointstowardboundbi1otherwise
  where *b*_*i*_ represents the bound.
- *Step tolerance*: at iteration *i* and for a set of parameters *x*_*i*_, if |*x*_*i*_ − *x*_*i*+1_| < 5e^−4^.

## Results

### Material parameters calibration via experimental data

After a total computational time of 10 days utilizing 20 AMD EPYC CPUs clocked at 3.1 GHz and 150 GB of RAM with parallelization, the optimization process converged for the three local solutions, created by the *MultiStart* option of MATLAB, due to step tolerance, and yielded different solutions in a total of 534 function evaluations, with the material parameters presented in Table 2.

**Fig 6:**
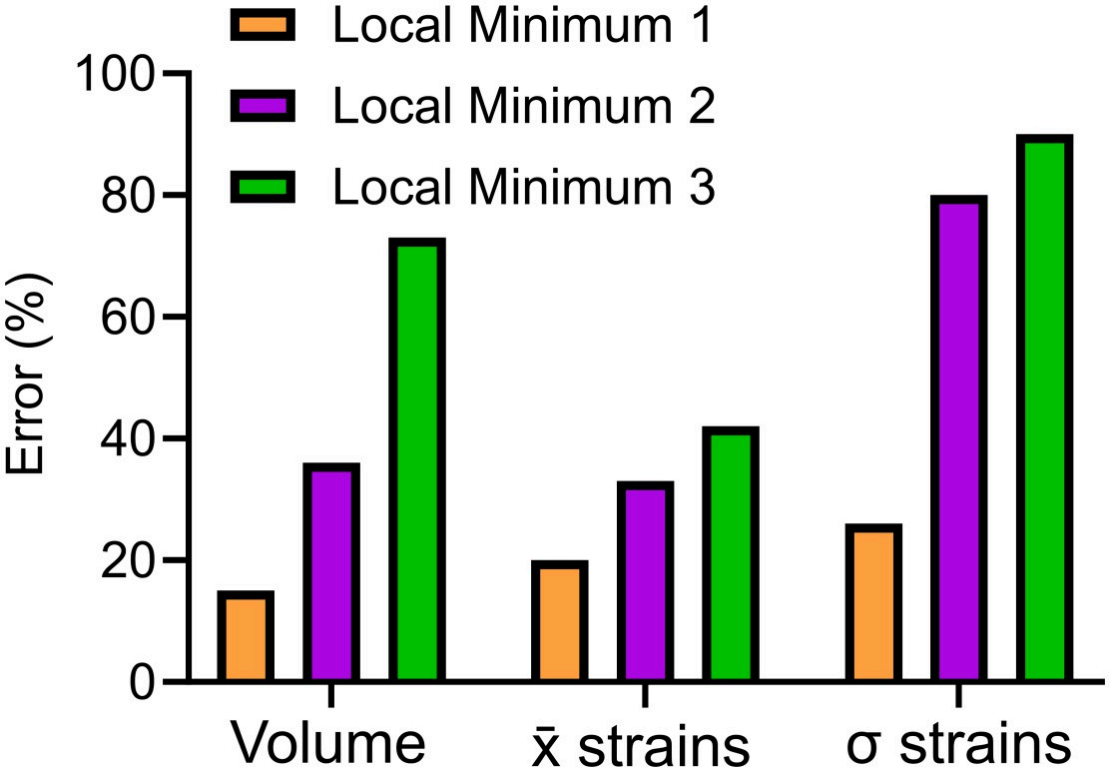
Errors between the experimental data and those obtained following the optimization process for three different solutions representing local minima. The relative error was computed using Eq 5 for the volumes, the average $\bar{\text{x}}$ strains, and the strain standard deviation (σ strains). Overall, the local minimum 1 provided a better solution.

A visual representation of the errors for various local minima can be found in Fig 6. We define a relative error related to the global volumes computed as follows:

$$Error_{Volume}\left(\%\right)=100\frac{\left|V_{exp}-V_{num}\right|}{V_{exp}}$$ (5)

where *V*_*num*_ and *V*_*exp*_ are respectively the model and the experimental volumes. The local strain errors for the average ($\bar{\text{x}}$) and standard deviation (σ) are analogously defined. When considering all three metrics of error (global pressure-volume response, local average strains and local strain distributions as the standard deviation), local minima 1 performs best.

**Table 2:** Initial starting points and converged calibrated parameters obtained from optimization. The MultiStart option was utilized to define a set of feasible starting points and search for local minima for each point [81]. The algorithm successfully converged to solutions for three of the local solutions. While the starting points 2 and 3 were suggested by the solver, starting point 1 was determined through a trial-and-error approach involving multiple simulations and a sensitivity analysis (S3 Appendix).

Parameters	*μ* (kPa)	*α*(−)	*ν*(−)	*k*_*UL*,*LL*,*UR*,*MR*,*LR*_ (10^−3^)(mm^2^.s^-1^)	*C*_10_ (kPa)	*λ*(−)
Starting Point 1	5.0	1.0	0.0	[10; 10; 10; 10; 10]	1.0	1.0e^−2^
**Local Minimum 1**	**2.8**	**3.0e** ^**−3**^	**0.0**	**[1.1; 0.7; 0.5; 8.2; 0.9]**	**0.1**	**0.7e** ^**−3**^
Starting Point 2	40	9.6	0.4	[79; 39; 69; 49; 12]	7.0	0.8
Local Minimum 2	4.0	4.3	0.2	[3.4; 7.3; 3.5; 10; 5.7]	0.4	1.3e^−2^
Starting Point 3	8.0	9.7	0.3	[65; 17; 95; 64; 95]	10	0.9
Local Minimum 3	7.8	4.3	0.1	[80; 84; 76; 66; 69]	1.1	4.0e^−3^

By converting the stiffness parameters thus obtained, and denoting $\mu_{parenchyma}^{i},\;\mu_{airways}^{i}$ and $\mu_{pleura}^{i}$ as the initial shear modulus of the local minimum number *i*, we found $\mu_{airways}^{1}=2\times C_{10}=0.2\;\text{kPa}$ and $\mu_{parenchyma}^{1}=2.8\;\text{kPa}$. The second local minimum exhibited similar stiffness for the parenchyma such that $\mu_{parenchyma}^{2}=4.0\;\text{kPa}$, and the airway material was stiffer with a shear modulus of $\mu_{airways}^{2}=0.8\;\text{kPa}$. The pleura material properties, driven by the parameter *k*, converged towards a low value for the local minima 1 and 3 with $\mu_{pleura}^{1}=2\times k\times C_{10}=1.7\;\text{Pa}$ and $\mu_{pleura}^{3}=10\;\text{Pa}$ compared to second local minima 2 $\mu_{pleura}^{2}=31\;\text{Pa}$. Finally, the third solution exhibited stiffer airways with $\mu_{airways}^{3}=2.2\;\text{kPa}$. The first local solution converged toward a high permeability, especially for the middle right lobe. The second solution presented permeabilities with the same order of magnitude for all five lobes while the third converged towards higher permeabilities in an attempt to reach the target strain values.

**Fig 7:**
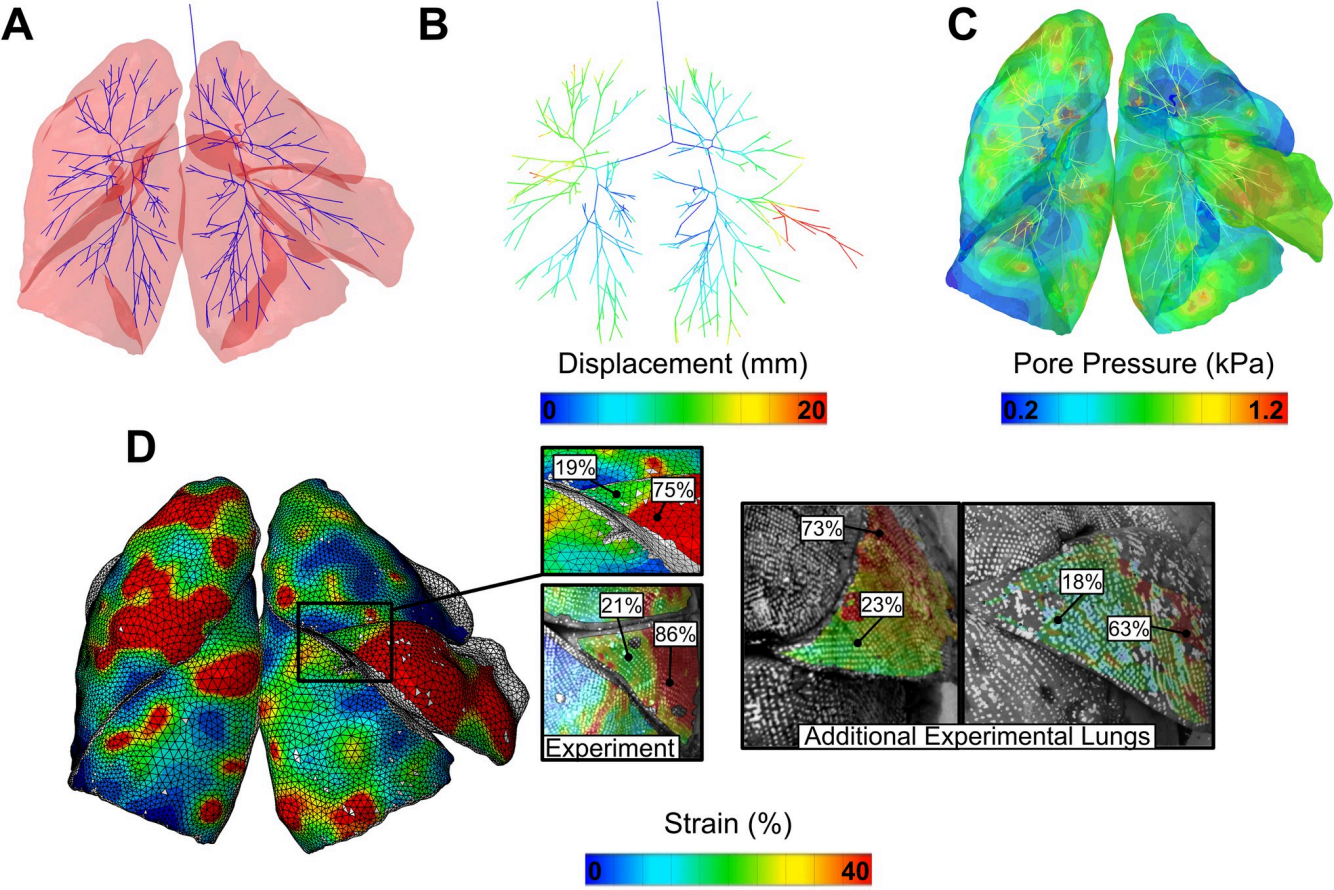
FE results after calibration. Translucent schematic **(A)** showing the relative airway placement within lobes where significant displacements are noted primarily in the distal airways **(B)**, while the trachea and the main bronchi remain stationary. Representation of pore pressure flow inside the lungs is shown in **(C)**. Major strains for the five lobes are visible in **(D)**, with an expanded view focusing on the middle right lobe, a region of interest that exhibits similar strain patterns to those observed in other human lungs with the same lobe configuration.

We proceed to focus on the material properties obtained from local minimum 1, since the overall error for the global and local strain averages and standard deviations perform best (refer to Fig 6). Fig 7 demonstrates end-inflation results. The translucent model (Fig 7A) with visible airway and resulting airway displacements due to the pressure flowing inside the lungs are depicted (Fig 7B). It can be observed that the pore pressure is predominantly concentrated at the ends of the airways, especially noticeable in the middle right and upper left lobes (Fig 7C). The resulting complex major strain patterns are illustrated in Fig 7D, with surface strains focused on the middle right lobe, in close correspondence with the experimental results for this particular lobe which tends to exhibit particularly lower strains at the corner, constrained by the larger adjacent lobes.

### Global calibration PV curve results

**Fig 8:**
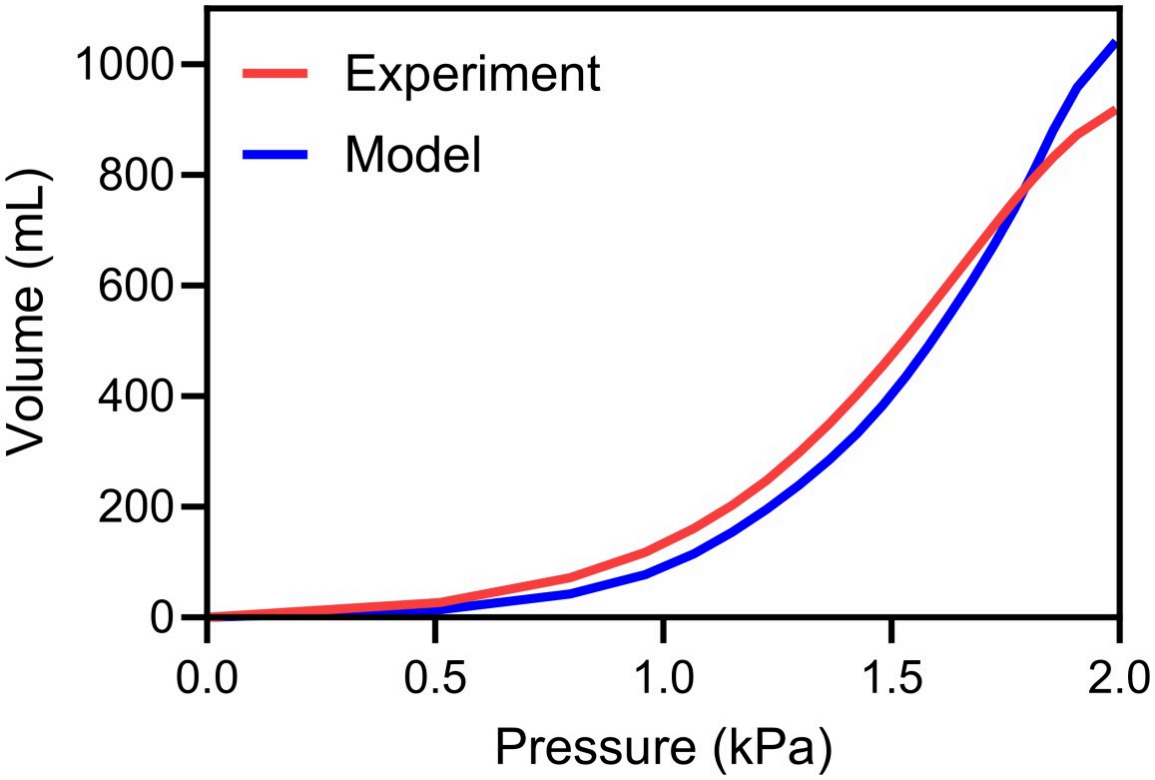
Comparison between the numerical model and the representative experimental PV curve during inflation, following calibration for local minimum 1. Early and late inflection characteristic response of the physiological curve is observed.

The PV curve obtained after optimization is depicted in Fig 8 (illustrated for local minima 1 as defined in Table 2). Using Eq 5, the averaged error obtained for local minima 1 stands at 15% showcasing the model’s proficiency in replicating the intricate shape of the PV curve. Notably the model is able to imitate the early and late inflection point characteristic of the physiological PV curve (Fig 1C).

### Local calibration of strains

**Fig 9:**
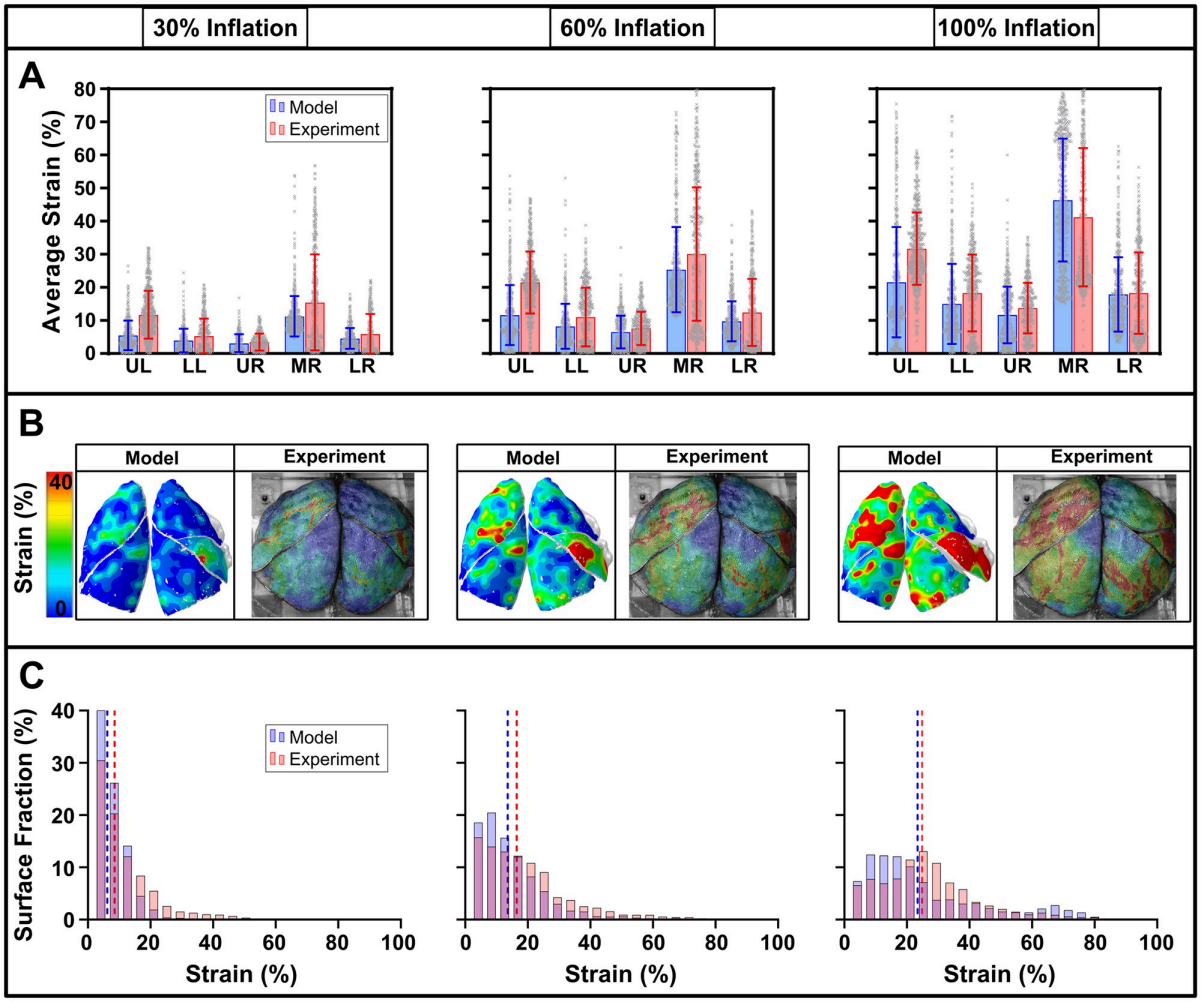
Post-calibration results of the model to subject-specific experiments. Comparisons between experimental data and the model results at inflation snapshots of 30%, 60%, and 100% of maximum pressure stages, respectively. **(A)** The regional distribution of strains is quantitatively contrasted between experiment (speckle points) and computational model (nodes) with 1000–2000 data points for each lobe shown with the average major strains and standard deviation bars. **(B)** illustrates the strain patterns and distributions across the lobes, while **(C)** quantifies the magnitude of strain distributions across the fractional surfaces of the entire lung where the vertical dashed lines represent the averages.

The strains were calibrated by comparing the average major strains and the standard deviation of strains for each of the five lobes at 30%, 60% and 100% inflation. The results for local minima 1 are displayed in Fig 9.

The FE model was able to generally reproduce the strain patterns and the distributions as defined by the standard deviation (Fig 9A), although it was unable to replicate the exact strain patterns at specific locations across the surface contours of the lung (Fig 9B). We observed similar higher strains for the upper left and middle right lobes, as well as an alternation of low and high strains within the same lobe, which are characteristics of human lung inflation. The quantity of strains and magnitudes, which are commonly populated at each inflation stage, are also well matched between the simulation and experiment (Fig 9C).

## Discussion

In this study we develop an IFEA framework to inform a generic structural model of the human lung using multi-scale and continuous experimental data from pressures, volumes, and strains. The pipeline is informed and verified using a cadaveric human lung, integrating Abaqus and MATLAB to well-represent the non-linear pulmonary pressure-volume evolution and complex strain contours associated with human breathing. The model’s ability to predict strains will be particularly useful in guiding clinicians during mechanical ventilation. It allows them to adjust parameters such as pressure, volume, and flow to ensure optimal support for patients while minimizing the risk of overstressing or damaging lung tissue [48]. We compare our optimized material parameters with those available in the literature and several key choices implemented in this study are discussed below, along with limitations for establishing the proof of concept framework.

The optimization process ran to search for three different local minima and computed the best overall solution corresponding to local minimum 1, as defined by the minimal error for global volume and local strain average and standard deviation (Eq 5). The resulting shear moduli of the parenchyma (*μ*_*parenchyma*_ = 2.8 kPa) aligns closely with the experimental shear modulus of 3.1±0.8 kPa (converted from reported elastic modulus with a fully compressible Poisson’s ratio [76]).

Unlike the parenchyma, the optimal airway shear modulus (*μ*_*airways*_ = 0.2 kPa) is low compared to experimental biaxial tests from human airways, which report a range of 2.1–4.3 kPa, (converted elastic modulus of the tracheal adventitia, mucosa, submucosa and trachealis muscle with an incompressible Poisson’s ratio [82]). The discrepancy may potentially be explained by (i) the material used for the airways (Neo-Hookean) may be too simplistic in light of previous complex constitutive models proposed [73], and (ii) the anisotropy of the airways may not be negligible [30,60].

In preliminary analyses, we conducted several simulations with higher airway stiffness values (above 10 kPa), which replicated the PV curves in the intricate shape but failed to sufficiently capture the local strain behavior. We also trialed different optimization loops and parameter bounds, but the solver consistently converged towards soft airways to ensure the strain distributions was achieved.

For the pleura stiffness, the best solutions were obtained when *μ*_*pleura*_ was low compared to the shear modulus of 2.4 kPa (where *k* = 1 for the fitted pleura stress-strain curve). Indeed, when *μ*_*pleura*_ is too high, it prevents the lobe from expanding more than 40% across our simulations and optimization tests, even if the parenchymal tissue is soft and the permeabilities are high, as was the case for the third local minima. Experimentally, the limited literature reports an shear modulus ranging from 0.1–0.8 kPa (converted from incompressible Poisson’s and measured Young’s modulus [83]), two orders of magnitude stiffer than the model. One source of discrepancy may be that experimental findings observed the pleura to be highly region dependent in contrast to the uniform stiffness value reported here [59]. Furthermore, the stiff pleura interfaces with the ultra-soft parenchyma and thus, isolated experimental tests on the pleura may measure an increased shear modulus, which does not accurately reflect the multi-constituent combinatory mechanics represented by the model.

Additionally, the reported optimized permeability parameters lack comparisons from the literature and inhibit evaluating the model’s performance and the physical validity of these values. Future experimental investigations on pulmonary permeabilities and pore pressures (Fig 7C) would enable and improve model validation capabilities.

While the strain averages and standard deviations were well captured by the model at increasing inflation stages, the location-specific strain contours were not precisely replicated. The intricate strain patterns are a result of airway positioning within the bulk parenchyma and localized inflation, along with the inherent properties of lung tissue itself and the lobe shapes; as such, this study’s inclusion of airways and intricate anatomical features, such as fissures between lobes, enriched the model’s physiological accuracy. On the other hand, given the inherent differences between the experimental and model geometries, striving for identical, location specific strain patterns is not feasible. Rather, our aim was thus restricted to establishing proof of concept and mechanical trends characteristic of human lungs to enable the generic model to reproduce these behaviors.

The method employed in this study was deemed direct, utilizing MATLAB’s built-in solution with the *lsqnonlin* function. However, depending on the complexity of the lungs, characterized by discrepancies in strains and high volumes, this optimization process could extend over several days. For one simulation, the computational time ranged between 20 min to more than 6 hours depending on the parameters. To mitigate this computational burden and potentially yield improved solutions, exploring alternative algorithms or solvers merits consideration. Another alternative is the use of reduced order techniques to reduce the model size while preserving its accuracy [84,85].

We conducted a comprehensive analysis of the mesh used in our model to determine the optimal type and quantity of elements. Increasing the mesh size to reduce computational costs revealed issues with contact modeling, and penetrations became problematic. Conversely, reducing the mesh size to improve the resolution prohibitively increased the computational cost. We defined the number of elements such that no penetration greater than 1 μm was recorded and ensured the geometry of the lungs was uncompromised.

While previous studies sought to generate solely patient-specific models [86–88], this study alternatively produces a generalizable breathing model to establish a numerical foundational tool that can improve our understanding of human lung function. Having established the proof of concept, this framework can now be further calibrated by multiple cadaveric human lungs, statistically tracking potential variabilities rooted in age, gender, pulmonary health state etc., for future in silico studies in order to gain unprecedented and clinically applicable insights regarding pulmonary physiology, and mirroring the impactful research trajectory of other organs in-silico studies [89–92].

### Limitations and future directions

As with all research, our study is subject to limitations primarily arising from computational cost restrictions or experimental logistics. For instance, strains used for validation are limited to the three dimensional surface atop the platform, trading off information regarding the internal structure of the lungs for beneficial continuous and directly linked global pressures and volumes and local tissue deformations [13]. This auxiliary data could support model improvements, however, may be deemed as a challenge considering it must integrate a scanner to obtain intraparenchymal measures or add cameras to track surface strains while interfacing with the dynamic PV ventilation system.

When calibrating the generic model, a challenge lied in defining appropriate metrics to ensure that the model captures the key characteristics of human lung inflation. For global behaviors, an acceptable metric within the literature is the matching of the changing lung volume and pressures [93,94]; however for strain, and in particular the surface strains unique to our experimental protocol, we defined both average and standard deviations as metrics for the magnitude and distributions of expected deformations for each lobe. Such a definition is limited since the metrics inherently rely on characteristics of the geometry of the lungs, which varies between individuals [95]. Reliable landmarks, other than easily decipherable lobes, means sectioning would be highly subjective (i.e. inferior lobe segments defined as medial basal, posterior basal, etc.). Defining appropriate metrics for capturing human lung breathing trends would benefit from objective segmenting methods to avoid capturing characteristics specific to individual patients’ geometric features.

Moreover, we do not currently account for anisotropy or local heterogeneity. Previous findings highlight the heterogeneous nature of the lung’s constituents [30,40,60]. In this study, we varied permeabilities between lobes to account for regional differences, but other parameters were not adjusted. Assigning different material parameters for each element to address local heterogeneity would have resulted in excessive computational costs and difficulties in finding appropriate solutions during the optimization process. Additionally, no studies have highlighted lobe-dependent variations in structural parameters compared to airflow-related parameters like permeabilities, which limits the justification for making these parameters lobe-dependent as well. While studies suggest that anisotropy may have a limited effect on strains [40], it may be more pertinent to incorporate material heterogeneity into our model as we previously mentioned. Moreover, incorporating anisotropy poses challenges, particularly in orientating elements within the lungs, although recent histological studies investigating fiber orientation could be used to inform our model [96,97].

Viscoelasticity, which could potentially aid in reaching the second inflection point in the PV curve as well as representing deflation, is not currently considered. Although incorporating viscoelasticity may increase the number of parameters and thus, the computational cost, utilizing differing hyperelastic material laws for lung components could mitigate this issue [98,99].

Regarding the parameters of this study, we used a specific method to calibrate the pleura’s stiffness by applying a factor λ to an average curve representing the experimental biaxial data. This approach has several limitations, as it does not account for the inherent heterogeneities of the pleura [59]. Future work could address this issue by conducting a more comprehensive analysis of the experiments and improving characterization by including more data points and recording the locations from multiple lungs to capture the stiffness gradient accurately.

Additionally, the permeabilities used in our model were considered constant, which does not fully reflect reality, as permeability is often linked to the shifting void ratio, especially for a complex organ like the lungs [100,101]. Further work is needed to develop an appropriate model for permeability while maintaining a reasonable quantity of newly introduced parameters in the optimization loop.

In this model, we modeled only 10 generations of airways. This represents a current limitation, as the human lung contains 23 generations [102]. The primary challenge lies in accurately capturing these geometric details from CT scans, which limits our ability to include additional branches [103].

Another crucial point in this study concerns the pressure application in our model. The pressure is applied at nodes near the ends of the airways, representing local expansion. However, these locations do not necessarily correspond to any specific physiological sites; the points do not represent alveoli or the exact ends of the branches, as the model only includes airways up to the 10th generation. While experimental evidence suggests that inflation involves regional popping [104], further work is needed to link the pressure points to physiological locations. In our study, we acknowledge the potential influence of gravity on the lung’s response, as suggested by previous research [35,105,106]. However, incorporating gravity into our model (although more realistic) would drastically extend the scope of this current study, which seeks to already introduce a multitude of novel factors. This current investigation establishes proof of concept and our future work will seek to incorporate gravitational effects.

Finally, the IFEA procedure contains several limitations that should be considered. The complexity of the model and the high computational cost constrain the full exploration of the parameter space. Moreover, the pipeline could be improved with additional experimental data to create a digital twin of a donor lung. However, the logistical challenges of obtaining such data (particularly MRI scans before lung excision) limit its application. We are actively working to adapt this approach for animal lungs, where more readily available data could facilitate improved personalized simulations. Lastly, while quick convergence during the IFEA procedure can be seen as an indicator of efficiency, it does not always guarantee a reliable model, and the initial point used in the optimization process affects convergence.

## Conclusion

In this proof of concept study we have devised an IFEA pipeline uniquely informed by continuous and multi-scale experimental data to construct the first 3D structural model of the inflating breathing lung. We successfully calibrate and validate the constructed framework using data from a cadaveric human lung. The resulting model effectively reproduces the global PV behavior and complex local deformations associated with lung expansion. Moving forward, this new framework holds promise for multiple applications, including the development of statistically substantiated human lungs and for exploring lung function across different species and pathologies.

## Supporting information

S1 Appendix Convexity assessment of the reduced polynomial function representing the pleura. (DOCX)

S2 Appendix Poroelastic formulation: Permeability and forchheimer’s law. (DOCX)

S3 Appendix Sensitivity analysis of the human lung model. (DOCX)

## References

[1] Ferkol T, Schraufnagel D. The Global Burden of Respiratory Disease. Ann Am Thorac Soc. 2014 Mar;11(3):404–6. doi: 10.1513/AnnalsATS.201311-405PS 24673696

[2] Xie M, Liu X, Cao X, Guo M, Li X. Trends in prevalence and incidence of chronic respiratory diseases from 1990 to 2017. Respir Res. 2020 Dec;21(1):49. doi: 10.1186/s12931-020-1291-8 32046720 PMC7014719

[3] Labaki WW, Han MK. Chronic respiratory diseases: a global view. Lancet Respir Med. 2020 Jun;8(6):531–3. doi: 10.1016/S2213-2600(20)30157-0 32526184 PMC8034823

[4] Yorgancioglu A, Khaltaev N, Bousquet J, Varghese C. The Global Alliance against Chronic Respiratory Diseases: journey so far and way ahead. Chin Med J (Engl). 2020 Jul 5;133(13):1513–5. doi: 10.1097/CM9.0000000000000851 32530880 PMC7386342

[5] Duan KI, Birger M, Au DH, Spece LJ, Feemster LC, Dieleman JL. Health Care Spending on Respiratory Diseases in the United States, 1996–2016. Am J Respir Crit Care Med. 2023 Jan 15;207(2):183–92. doi: 10.1164/rccm.202202-0294OC 35997678 PMC9893322

[6] D’Amato G, Pawankar R, Vitale C, Lanza M, Molino A, Stanziola A, et al. Climate Change and Air Pollution: Effects on Respiratory Allergy. Allergy Asthma Immunol Res. 2016;8(5):391. doi: 10.4168/aair.2016.8.5.391 27334776 PMC4921692

[7] Boehm A, Pizzini A, Sonnweber T, Loeffler-Ragg J, Lamina C, Weiss G, et al. Assessing global COPD awareness with Google Trends. Eur Respir J. 2019 Jun;53(6):1900351. doi: 10.1183/13993003.00351-2019 31097517

[8] Qureshi NQ, Mufarrih SH, Bloomfield GS, Tariq W, Almas A, Mokdad AH, et al. Disparities in Cardiovascular Research Output and Disease Outcomes among High-, Middle- and Low-Income Countries–An Analysis of Global Cardiovascular Publications over the Last Decade (2008–2017). Glob Heart. 2021 Jan 18;16(1):4. doi: 10.5334/gh.815 33598384 PMC7845477

[9] Akiki V, Troussard X, Metges J, Devos P. Global trends in oncology research: A mixed-methods study of publications and clinical trials from 2010 to 2019. Cancer Rep. 2023 Jan;6(1):e1650. doi: 10.1002/cnr2.1650 35689556 PMC9875665

[10] Elsheikh A, Wang D. Numerical modelling of corneal biomechanical behaviour. Comput Methods Biomech Biomed Engin. 2007 Apr;10(2):85–95. doi: 10.1080/10255840600976013 18651274

[11] Badrou A, Tardif N, Chaudet P, Lescanne N, Szewczyk J, Blanc R, et al. Simulation of multi-curve active catheterization for endovascular navigation to complex targets. J Biomech. 2022 Jul;140:111147. doi: 10.1016/j.jbiomech.2022.111147 35667147

[12] Xu Y, Yamashiro T, Moriya H, Tsubakimoto M, Nagatani Y, Matsuoka S, et al. Strain measurement on four-dimensional dynamic-ventilation CT: quantitative analysis of abnormal respiratory deformation of the lung in COPD. Int J Chron Obstruct Pulmon Dis. 2018 Dec;Volume 14:65–72. doi: 10.2147/COPD.S183740 30587962 PMC6305131

[13] Mariano CA, Sattari S, Quiros KAM, Nelson TM, Eskandari M. Examining lung mechanical strains as influenced by breathing volumes and rates using experimental digital image correlation. Respir Res. 2022 Dec;23(1):92. doi: 10.1186/s12931-022-01999-7 35410291 PMC8999998

[14] Farkas Á, Lizal F, Elcner J, Jedelsky J, Jicha M. Numerical simulation of fibre deposition in oral and large bronchial airways in comparison with experiments. J Aerosol Sci. 2019 Oct;136:1–14.

[15] Mondal J, Khajanchi S. Mathematical modeling and optimal intervention strategies of the COVID-19 outbreak. Nonlinear Dyn. 2022 Jul;109(1):177–202. doi: 10.1007/s11071-022-07235-7 35125654 PMC8801045

[16] Medrek M, Pastuszak Z. Numerical simulation of the novel coronavirus spreading. Expert Syst Appl. 2021 Mar;166:114109. doi: 10.1016/j.eswa.2020.114109 33078047 PMC7557303

[17] Salati M, Brunelli A. Risk Stratification in Lung Resection. Curr Surg Rep. 2016 Nov;4(11):37. doi: 10.1007/s40137-016-0158-x 27730011 PMC5030224

[18] Radhakrishnan H, Kassinos S. CFD modeling of turbulent flow and particle deposition in human lungs. In: 2009 Annual International Conference of the IEEE Engineering in Medicine and Biology Society [Internet]. Minneapolis, MN: IEEE; 2009 [cited 2024 Mar 19]. p. 2867–70. http://ieeexplore.ieee.org/document/5333102/. 10.1109/IEMBS.2009.5333102 19963784

[19] Nowak N, Kakade PP, Annapragada AV. Computational Fluid Dynamics Simulation of Airflow and Aerosol Deposition in Human Lungs. Ann Biomed Eng. 2003 Apr;31(4):374–90. doi: 10.1114/1.1560632 12723679

[20] Aliboni L, Pennati F, Sarti M, Iorio V, Carrinola R, Palleschi A, et al. Computational Fluid Dynamics (CFD) Analysis of Subject-specific Bronchial Tree Models in Lung Cancer Patients. In: 2021 43rd Annual International Conference of the IEEE Engineering in Medicine & Biology Society (EMBC) [Internet]. Mexico: IEEE; 2021 [cited 2024 Mar 19]. p. 4281–4. https://ieeexplore.ieee.org/document/9629765/. 10.1109/EMBC46164.2021.9629765 34892168

[21] Poorbahrami K, Oakes JM. Regional flow and deposition variability in adult female lungs: A numerical simulation pilot study. Clin Biomech. 2019 Jun;66:40–9. doi: 10.1016/j.clinbiomech.2017.12.014 29395490

[22] Caucha LJ, Frei S, Rubio O. Finite element simulation of fluid dynamics and CO_2_ gas exchange in the alveolar sacs of the human lung. Comput Appl Math. 2018 Nov;37(5):6410–32.

[23] Filipovic N. Machine Learning and Finite Element Methods in Modeling of COVID-19 Spread. In: Tsihrintzis GA, Virvou M, Esposito A, Jain LC, editors. Advances in Assistive Technologies [Internet]. Cham: Springer International Publishing; 2022 [cited 2024 Mar 19]. p. 43–69. (Learning and Analytics in Intelligent Systems; vol. 28). https://link.springer.com/10.1007/978-3-030-87132-1_4.

[24] Hayati H, Feng Y, Chen X, Kolewe E, Fromen C. Prediction of transport, deposition, and resultant immune response of nasal spray vaccine droplets using a CFPD-HCD model in a 6-year-old upper airway geometry to potentially prevent COVID-19. Exp Comput Multiph Flow. 2023 Sep;5(3):272–89. doi: 10.1007/s42757-022-0145-7 36694695 PMC9851113

[25] Werner R, Ehrhardt J, Schmidt R, Handels H. Patient-specific finite element modeling of respiratory lung motion using 4D CT image data. Med Phys. 2009 May;36(5):1500–11. doi: 10.1118/1.3101820 19544766

[26] Hasani M, Razaghi R, Hassani K, Rahmati SM, Tehrani P, Karimi A. A patient-specific finite element model of the smoker’s lung during breathing. Proc Inst Mech Eng Part E J Process Mech Eng. 2021 Aug;235(4):879–86.

[27] Bayliss LE, Robertson GW. THE VISCO-ELASTIC PROPERTIES OF THE LUNGS. Q J Exp Physiol Cogn Med Sci. 1939 Mar 22;29(1):27–47.

[28] Amelon R, Cao K, Ding K, Christensen GE, Reinhardt JM, Raghavan ML. Three-dimensional characterization of regional lung deformation. J Biomech. 2011 Sep;44(13):2489–95. doi: 10.1016/j.jbiomech.2011.06.009 21802086 PMC3443473

[29] Hurtado DE, Villarroel N, Andrade C, Retamal J, Bugedo G, Bruhn A. Spatial patterns and frequency distributions of regional deformation in the healthy human lung. Biomech Model Mechanobiol. 2017 Aug;16(4):1413–23. doi: 10.1007/s10237-017-0895-5 28315975

[30] Eskandari M, Arvayo AL, Levenston ME. Mechanical properties of the airway tree: heterogeneous and anisotropic pseudoelastic and viscoelastic tissue responses. J Appl Physiol. 2018 Sep 1;125(3):878–88. doi: 10.1152/japplphysiol.00090.2018 29745796

[31] Li M, Castillo E, Zheng XL, Luo HY, Castillo R, Wu Y, et al. Modeling lung deformation: A combined deformable image registration method with spatially varying Young’s modulus estimates: Modeling lung deformation. Med Phys. 2013 Jul 3;40(8):081902. 23927316 10.1118/1.4812419 PMC3716779

[32] Al-Mayah A, Moseley J, Brock KK. Contact surface and material nonlinearity modeling of human lungs. Phys Med Biol. 2008 Jan 7;53(1):305–17. doi: 10.1088/0031-9155/53/1/022 18182705

[33] Neelakantan S, Xin Y, Gaver DP, Cereda M, Rizi R, Smith BJ, et al. Computational lung modelling in respiratory medicine. J R Soc Interface. 2022 Jun;19(191):20220062. doi: 10.1098/rsif.2022.0062 35673857 PMC9174712

[34] Wall WA, Rabczuk T. Fluid–structure interaction in lower airways of CT-based lung geometries. Int J Numer Methods Fluids. 2008 Jun 20;57(5):653–75.

[35] Seyfi B, Santhanam AP, Ilegbusi OJ. A Biomechanical Model of Human Lung Deformation Utilizing Patient-Specific Elastic Property. J Cancer Ther. 2016;07(06):402–15.

[36] Hurtado DE, Avilés-Rojas N, Concha F. Multiscale modeling of lung mechanics: From alveolar microstructure to pulmonary function. J Mech Phys Solids. 2023 Oct;179:105364.

[37] Patte C, Genet M, Chapelle D. A quasi-static poromechanical model of the lungs. Biomech Model Mechanobiol. 2022 Apr;21(2):527–51. doi: 10.1007/s10237-021-01547-0 35072891

[38] Laville C, Fetita C, Gille T, Brillet PY, Nunes H, Bernaudin JF, et al. Comparison of optimization parametrizations for regional lung compliance estimation using personalized pulmonary poromechanical modeling. Biomech Model Mechanobiol. 2023 Oct;22(5):1541–54. doi: 10.1007/s10237-023-01691-9 36913005 PMC10009868

[39] Sarabia-Vallejos MA, Zuñiga M, Hurtado DE. The role of three-dimensionality and alveolar pressure in the distribution and amplification of alveolar stresses. Sci Rep. 2019 Jun 19;9(1):8783. doi: 10.1038/s41598-019-45343-4 31217511 PMC6584652

[40] Maghsoudi-Ganjeh M, Mariano CA, Sattari S, Arora H, Eskandari M. Developing a Lung Model in the Age of COVID-19: A Digital Image Correlation and Inverse Finite Element Analysis Framework. Front Bioeng Biotechnol. 2021 Oct 26;9:684778. doi: 10.3389/fbioe.2021.684778 34765590 PMC8576180

[41] Sattari S, Mariano CA, Vittalbabu S, Velazquez JV, Postma J, Horst C, et al. Introducing a Custom-Designed Volume-Pressure Machine for Novel Measurements of Whole Lung Organ Viscoelasticity and Direct Comparisons Between Positive- and Negative-Pressure Ventilation. Front Bioeng Biotechnol. 2020 Oct 21;8:578762. doi: 10.3389/fbioe.2020.578762 33195138 PMC7643401

[42] Mariano CA, Sattari S, Maghsoudi-Ganjeh M, Tartibi M, Lo DD, Eskandari M. Novel Mechanical Strain Characterization of Ventilated ex vivo Porcine and Murine Lung using Digital Image Correlation. Front Physiol. 2020 Dec 4;11:600492. doi: 10.3389/fphys.2020.600492 33343395 PMC7746832

[43] Eskandari M, Mariano CA, Sattari S, Nelson TM, Anduaga KAM. Human Versus Porcine Localized Strain Mechanics. In: D109 AIRWAY OF INTEREST: EPITHELIAL AND SMOOTH MUSCLE FUNCTION IN HEALTH AND DISEASE [Internet]. American Thoracic Society; 2022 [cited 2024 Jul 8]. p. A5502–A5502. https://www.atsjournals.org/doi/10.1164/ajrccm-conference.2022.205.1_MeetingAbstracts.A5502.

[44] Sattari S, Mariano CA, Kuschner WG, Taheri H, Bates JHT, Eskandari M. Positive- and Negative-Pressure Ventilation Characterized by Local and Global Pulmonary Mechanics. Am J Respir Crit Care Med. 2023 Mar 1;207(5):577–86. doi: 10.1164/rccm.202111-2480OC 36194677 PMC10870900

[45] Quiros KAM, Nelson TM, Ulu A, Dominguez EC, Biddle TA, Lo DD, et al. A Comparative Study of Ex-Vivo Murine Pulmonary Mechanics Under Positive- and Negative-Pressure Ventilation. Ann Biomed Eng. 2024 Feb;52(2):342–54. doi: 10.1007/s10439-023-03380-1 37906375 PMC10808462

[46] Eskandari M, Sattari S, Quiros K. The Role of Interspecies Variability on Positive- Versus Negative-pressure Ventilation Mechanics. In: C72 HOUSE OF ARDS.AND MECHANICAL VENTILATORY SUPPORT [Internet]. American Thoracic Society; 2023 [cited 2024 Dec 3]. p. A5776–A5776. https://www.atsjournals.org/doi/10.1164/ajrccm-conference.2023.207.1_MeetingAbstracts.A5776.

[47] Nelson TM, Quiros KAM, Dominguez EC, Ulu A, Nordgren TM, Eskandari M. Diseased and healthy murine local lung strains evaluated using digital image correlation. Sci Rep. 2023 Mar 20;13(1):4564. doi: 10.1038/s41598-023-31345-w 36941463 PMC10026788

[48] Nelson TM, Quiros KAM, Mariano CA, Sattari S, Ulu A, Dominguez EC, et al. Associating local strains to global pressure–volume mouse lung mechanics using digital image correlation. Physiol Rep. 2022 Oct;10(19):e15466. doi: 10.14814/phy2.15466 36207795 PMC9547081

[49] Ligas JR. Pleural Mechanics. In: Epstein MAF, Ligas JR, editors. Respiratory Biomechanics [Internet]. New York, NY: Springer New York; 1990 [cited 2024 Sep 2]. p. 44–51. http://link.springer.com/10.1007/978-1-4612-3452-4_5.

[50] Jiang Y, Xiong Z, Zhao W, Tian D, Zhang Q, Li Z. Pathological components and CT imaging analysis of the area adjacent pleura within the pure ground-glass nodules with pleural deformation in invasive lung adenocarcinoma. BMC Cancer. 2022 Sep 6;22(1):958. doi: 10.1186/s12885-022-10043-2 36068487 PMC9447332

[51] Computing A. HyperMesh Reference Manual -: Version 2.1 [Internet]. Altair Computing, Incorporated; 1997. https://books.google.com/books?id=uTRUYAAACAAJ.

[52] Tzeng Y, Hoffman E, Cook-Granroth J, Maurer R, Shah N, Mansour J, et al. Comparison of airway diameter measurements from an anthropomorphic airway tree phantom using hyperpolarized ^3^He MRI and high-resolution computed tomography. Magn Reson Med. 2007 Sep;58(3):636–42. 17763351 10.1002/mrm.21285 PMC2943874

[53] Fung YC. Biomechanics: Mechanical Properties of Living Tissues. Springer New York; 2013.

[54] Kroon M. A constitutive framework for modelling thin incompressible viscoelastic materials under plane stress in the finite strain regime. Mech Time-Depend Mater. 2011 Nov;15(4):389–406.

[55] Dassault Systemes Simulia, Inc. ABAQUS (2023) Analysis User’s Manual, Version 2023.

[56] Storåkers B. On material representation and constitutive branching in finite compressible elasticity. J Mech Phys Solids. 1986 Jan;34(2):125–45.

[57] Yamada Y, Yamada M, Chubachi S, Yokoyama Y, Matsuoka S, Tanabe A, et al. Comparison of inspiratory and expiratory lung and lobe volumes among supine, standing, and sitting positions using conventional and upright CT. Sci Rep. 2020 Oct 1;10(1):16203. doi: 10.1038/s41598-020-73240-8 33004894 PMC7530723

[58] Valipour A, Kramer MR, Stanzel F, Kempa A, Asadi S, Fruchter O, et al. Physiological Modeling of Responses to Upper Versus Lower Lobe Lung Volume Reduction in Homogeneous Emphysema. Front Physiol [Internet]. 2012 [cited 2024 Sep 5];3. Available from: http://journal.frontiersin.org/article/10.3389/fphys.2012.00387/abstract. 23060811 10.3389/fphys.2012.00387 PMC3461642

[59] Ramirez GO, Mariano CA, Carter D, Eskandari M. Visceral Pleura Mechanics: Characterization of Human, Pig, and Rat Lung Material Properties. Acta Biomater. 2024 Sep;S1742706124005191. doi: 10.1016/j.actbio.2024.09.003 39251049

[60] Sattari S, Mariano CA, Eskandari M. Biaxial mechanical properties of the bronchial tree: Characterization of elasticity, extensibility, and energetics, including the effect of strain rate and preconditioning. Acta Biomater. 2023 Jan;155:410–22. doi: 10.1016/j.actbio.2022.10.047 36328122

[61] Mariano CA. Human Airways Tissue Biaxial Tensile Mechanics. American Society of Mechanical Engineers Bioengineering Division: Summer of Biomechanics Bioengineering Biotransport Conference; 2024 Jun 11.

[62] Nelson TM, Mariano CA, Ramirez GO, Badrou A, Quiros KAM, Shankel M, et al. Lung Mechanics: Material Characterization of Pulmonary Constituents for an Experimentally Informed Computational Pipeline. Curr Protoc. 2024 Sep;4(9):e70001. doi: 10.1002/cpz1.70001 39240156

[63] Dietrich CF, Mathis G, Cui XW, Ignee A, Hocke M, Hirche TO. Ultrasound of the Pleurae and Lungs. Ultrasound Med Biol. 2015 Feb;41(2):351–65. doi: 10.1016/j.ultrasmedbio.2014.10.002 25592455

[64] Vial MR, Interventional Pulmonology Unit, Clinica Alemana de Santiago-Universidad del Desarrollo, Chile, Grosu HB, Department of Pulmonary Medicine, The University of Texas MD Anderson Cancer Center, Houston, TX, US. Practice Pearls for Performing Pleural Ultrasound with Focus on Pleural Effusion and Pleural Thickening. US Respir Pulm Dis. 2017;12(02):23.

[65] Chatelin S, Oudry J, Périchon N, Sandrin L, Allemann P, Soler L, et al. In vivo liver tissue mechanical properties by transient elastography: Comparison with dynamic mechanical analysis. Biorheology. 2011;48(2):75–88. doi: 10.3233/BIR-2011-0584 21811013

[66] Galloy AE, Reinhardt JM, Raghavan ML. Role of lung lobar sliding on parenchymal distortion during breathing. J Appl Physiol. 2023 Sep 1;135(3):534–41. doi: 10.1152/japplphysiol.00631.2022 37439240 PMC10538991

[67] Lee GC, Tseng NT, Yuan YM. Finite element modeling of lungs including interlobar fissures and the heart cavity. J Biomech. 1983 Jan;16(9):679–90. doi: 10.1016/0021-9290(83)90078-7 6643540

[68] Lumb AB. Functional Anatomy of the Respiratory Tract. In: Nunn’s Applied Respiratory Physiology [Internet]. Elsevier; 2017 [cited 2024 Apr 16]. p. 3–16.e1. https://linkinghub.elsevier.com/retrieve/pii/B9780702062940000010.

[69] Ma B, Smith BJ, Bates JHT. Resistance to alveolar shape change limits range of force propagation in lung parenchyma. Respir Physiol Neurobiol. 2015 Jun;211:22–8. doi: 10.1016/j.resp.2015.03.004 25812796 PMC4406872

[70] Nelson TM, Quiros KAM, Dominguez EC, Ulu A, Nordgren TM, Nair MG, et al. Healthy and diseased tensile mechanics of mouse lung parenchyma. Results Eng. 2024 Jun;22:102169.

[71] Grossman E, Grossman A, Schein M, Zimlichman R, Gavish B. Breathing-control lowers blood pressure. J Hum Hypertens. 2001 Apr 1;15(4):263–9. doi: 10.1038/sj.jhh.1001147 11319675

[72] Russo MA, Santarelli DM, O’Rourke D. The physiological effects of slow breathing in the healthy human. Breathe. 2017 Dec;13(4):298–309. doi: 10.1183/20734735.009817 29209423 PMC5709795

[73] Eskandari M, Nordgren TM, O’Connell GD. Mechanics of pulmonary airways: Linking structure to function through constitutive modeling, biochemistry, and histology. Acta Biomater. 2019 Oct;97:513–23. doi: 10.1016/j.actbio.2019.07.020 31330329 PMC7462120

[74] Masri C, Chagnon G, Favier D, Sartelet H, Girard E. Experimental characterization and constitutive modeling of the biomechanical behavior of male human urethral tissues validated by histological observations. Biomech Model Mechanobiol. 2018 Aug;17(4):939–50. doi: 10.1007/s10237-018-1003-1 29380159

[75] Mariano CA, Sattari S, Ramirez GO, Eskandari M. Effects of tissue degradation by collagenase and elastase on the biaxial mechanics of porcine airways. Respir Res. 2023 Apr 8;24(1):105. doi: 10.1186/s12931-023-02376-8 37031200 PMC10082978

[76] Polio SR, Kundu AN, Dougan CE, Birch NP, Aurian-Blajeni DE, Schiffman JD, et al. Cross-platform mechanical characterization of lung tissue. Dague E, editor. PLOS ONE. 2018 Oct 17;13(10):e0204765. doi: 10.1371/journal.pone.0204765 30332434 PMC6192579

[77] Henne E, Anderson JC, Lowe N, Kesten S. Comparison of human lung tissue mass measurements from Ex Vivo lungs and high resolution CT software analysis. BMC Pulm Med. 2012 Dec;12(1):18. doi: 10.1186/1471-2466-12-18 22584018 PMC3499450

[78] Walotek K, Bzówka J, Ciołczyk A. Examples of the Use of the ARAMIS 3D Measurement System for the Susceptibility to Deformation Tests for the Selected Mixtures of Coal Mining Wastes. Sensors. 2021 Jul 5;21(13):4600. doi: 10.3390/s21134600 34283159 PMC8272077

[79] Milligan GW, Cooper MC. A study of standardization of variables in cluster analysis. J Classif. 1988 Sep;5(2):181–204.

[80] Coleman TF, Li Y. An Interior Trust Region Approach for Nonlinear Minimization Subject to Bounds. SIAM J Optim. 1996 May;6(2):418–45.

[81] The MathWorks Inc. Optimization Toolbox version: 9.4 (R2022b) [Internet]. Natick, Massachusetts, United States: The MathWorks Inc.; 2022. https://www.mathworks.com.

[82] Teng Z, Trabelsi O, Ochoa I, He J, Gillard JH, Doblare M. Anisotropic material behaviours of soft tissues in human trachea: An experimental study. J Biomech. 2012 Jun;45(9):1717–23. doi: 10.1016/j.jbiomech.2012.04.002 22534565

[83] Eskandari M, Ramirez GO, Mariano CA. Investigating Visceral Pleura Mechanics to Improve Biomimetic Surgical Adhesives Design. In: C73 PRECLINICAL MODELING OF PULMONARY INFLAMMATION AND EMPHYSEMA [Internet]. American Thoracic Society; 2024 [cited 2024 Jul 8]. p. A6449–A6449. https://www.atsjournals.org/doi/10.1164/ajrccm-conference.2024.209.1_MeetingAbstracts.A6449.

[84] Badías A, González D, Alfaro I, Chinesta F, Cueto E. An introduction to model order reduction techniques. In: Reduced Order Models for the Biomechanics of Living Organs [Internet]. Elsevier; 2023 [cited 2024 Mar 30]. p. 3–21. https://linkinghub.elsevier.com/retrieve/pii/B9780323899673000032.

[85] Badrou A, Bel-Brunon A, Hamila N, Tardif N, Gravouil A. Reduced order modeling of an active multi-curve guidewire for endovascular surgery. Comput Methods Biomech Biomed Engin. 2020 Oct 19;23(sup1):S23–4.

[86] Han L, Dong H, McClelland JR, Han L, Hawkes DJ, Barratt DC. A hybrid patient-specific biomechanical model based image registration method for the motion estimation of lungs. Med Image Anal. 2017 Jul;39:87–100. doi: 10.1016/j.media.2017.04.003 28458088

[87] Ladjal H, Beuve M, Giraud P, Shariat B. Towards Non-Invasive Lung Tumor Tracking Based on Patient Specific Model of Respiratory System. IEEE Trans Biomed Eng. 2021 Sep;68(9):2730–40. doi: 10.1109/TBME.2021.3053321 33476262

[88] Sun Q, Zhou C, Chase JG. Parameter updating of a patient-specific lung mechanics model for optimising mechanical ventilation. Biomed Signal Process Control. 2020 Jul;60:102003.

[89] Miller K. Constitutive model of brain tissue suitable for finite element analysis of surgical procedures. J Biomech. 1999 May;32(5):531–7. doi: 10.1016/s0021-9290(99)00010-x 10327007

[90] Gasser TC, Auer M, Labruto F, Swedenborg J, Roy J. Biomechanical Rupture Risk Assessment of Abdominal Aortic Aneurysms: Model Complexity versus Predictability of Finite Element Simulations. Eur J Vasc Endovasc Surg. 2010 Aug;40(2):176–85. doi: 10.1016/j.ejvs.2010.04.003 20447844

[91] Baillargeon B, Rebelo N, Fox DD, Taylor RL, Kuhl E. The Living Heart Project: A robust and integrative simulator for human heart function. Eur J Mech—ASolids. 2014 Nov;48:38–47. doi: 10.1016/j.euromechsol.2014.04.001 25267880 PMC4175454

[92] Schileo E, Taddei F, Malandrino A, Cristofolini L, Viceconti M. Subject-specific finite element models can accurately predict strain levels in long bones. J Biomech. 2007;40(13):2982–9. doi: 10.1016/j.jbiomech.2007.02.010 17434172

[93] Axe JR, Abbrecht PH. Analysis of the pressure-volume relationship of excised lungs. Ann Biomed Eng. 1985 Mar;13(2):101–17. doi: 10.1007/BF02584233 4003874

[94] Tepper JS, Costa DL. Methods, Measurements, and Interpretation of Animal Lung Function in Health and Disease. In: Comparative Biology of the Normal Lung [Internet]. Elsevier; 2015 [cited 2024 Jul 12]. p. 305–51. https://linkinghub.elsevier.com/retrieve/pii/B9780124045774000175.

[95] George BM, Nayak SB, Marpalli S. Morphological variations of the lungs: a study conducted on Indian cadavers. Anat Cell Biol. 2014;47(4):253. doi: 10.5115/acb.2014.47.4.253 25548723 PMC4276899

[96] Lemos M, Pozo RMK, Montes GS, Saldiva PHN. Organization of collagen and elastic fibers studied in stretch preparations of whole mounts of human visceral pleura. Ann Anat—Anat Anz. 1997 Oct;179(5):447–52. doi: 10.1016/S0940-9602(97)80048-9 9341952

[97] Polzer S, Thompson S, Vittalbabu S, Ulu A, Carter D, Nordgren T, et al. MATLAB-Based Algorithm and Software for Analysis of Wavy Collagen Fibers. Microsc Microanal. 2023 Dec 21;29(6):2108–26. doi: 10.1093/micmic/ozad117 37992253

[98] Chatelin S, Deck C, Willinger R. An anisotropic viscous hyperelastic constitutive law for brain material finite-element modeling. J Biorheol. 2013 Nov;27(1–2):26–37.

[99] Panda SK, Buist ML. A finite nonlinear hyper-viscoelastic model for soft biological tissues. J Biomech. 2018 Mar;69:121–8. doi: 10.1016/j.jbiomech.2018.01.025 29397112

[100] Simon BR, Kaufmann MV, McAfee MA, Baldwin AL. Porohyperelastic Finite Element Analysis of Large Arteries Using ABAQUS. J Biomech Eng. 1998 Apr 1;120(2):296–8. doi: 10.1115/1.2798315 10412393

[101] Mow VC, Holmes MH, Michael Lai W. Fluid transport and mechanical properties of articular cartilage: A review. J Biomech. 1984 Jan;17(5):377–94. doi: 10.1016/0021-9290(84)90031-9 6376512

[102] Weibel ER, Gomez DM. Architecture of the Human Lung: Use of quantitative methods establishes fundamental relations between size and number of lung structures. Science. 1962 Aug 24;137(3530):577–85. 14005590 10.1126/science.137.3530.577

[103] Pu J, Gu S, Liu S, Zhu S, Wilson D, Siegfried JM, et al. CT based computerized identification and analysis of human airways: A review. Med Phys. 2012 May;39(5):2603–16. doi: 10.1118/1.4703901 22559631 PMC3344883

[104] Quiros KAM, Nelson TM, Sattari S, Mariano CA, Ulu A, Dominguez EC, et al. Mouse lung mechanical properties under varying inflation volumes and cycling frequencies. Sci Rep. 2022 May 2;12(1):7094. doi: 10.1038/s41598-022-10417-3 35501363 PMC9059689

[105] Prisk GK. Microgravity and the respiratory system. Eur Respir J. 2014 May 1;43(5):1459–71. doi: 10.1183/09031936.00001414 24603820

[106] Shi L, Herrmann J, Bou Jawde S, Bates JHT, Nia HT, Suki B. Modeling the influence of gravity and the mechanical properties of elastin and collagen fibers on alveolar and lung pressure–volume curves. Sci Rep. 2022 Jul 19;12(1):12280. doi: 10.1038/s41598-022-16650-0 35853981 PMC9294799

