# Supplementary material for: Towards constructing a generalized structural 3D breathing human lung model based on experimental volumes, pressures, and strains
Source: PLoS Comput Biol. 2025 Jan 13;21(1):e1012680. doi: 10.1371/journal.pcbi.1012680 (PMC11729960; doi:10.1371/journal.pcbi.1012680)

**Convexity Assessment of the Reduced Polynomial Function Representing the Pleura**

To model the pleura with membrane elements and assumed incompressibility, we used a form of the strain energy function $U_{RP}$ known as reduced polynomial model, defined as follows:

$$U_{RP}= \sum_{i=1}^{n} {C_{i0}\left( \bar{I_{1}}-3 \right)}^{i}$$

where $C_{10}$ is a material parameter related to the stiffness of the tissue and $\bar{I_{1}}$ is the first invariant of the right Cauchy-Green deformation tensor. We want to ensure that the material is convex with respect to the Green Lagrange strain tensor $\text{E}$. Specifically, the second derivative of the strain energy function with respect to $\bar{I_{1}}$ needs to be positive over the range of $\bar{I_{1}}$​ to guarantee the convexity of the reduced polynomial function used to represent the pleura's behavior, since $\bar{I_{1}}=3+2\text{tr}(\text{E})$.

The second derivative of $U_{RP}$ with respect to $\bar{I_{1}}$​ is computed as:

$$\frac{d^{2}U_{RP}}{d^{2}\bar{I_{1}}}=\sum_{i=2}^{5} C_{i}\times i\times(i-1)\times{(\bar{I_{1}}-3)}^{i-2}$$

The material is assumed to be incompressible. Therefore, for the uniaxial tension with a stretch $\lambda$ where the material is assumed incompressible has a first invariant $\bar{I_{1}}=\frac{\lambda^{2}}{2}+\frac{2}{\lambda}-\frac{3}{2}$​. For a biaxial test, we have $\bar{I_{1}}=\lambda^{2}+\frac{1}{\lambda^{4}}-\frac{3}{2}$.

Thus $\bar{I_{1}}\in\left[ 0;5 \right]$ to cover (including in compression) between 0% and 100% of strain. A plot of the second derivative of the strain energy function with respect to $\bar{I_{1}}$ (see Figure below) demonstrates that the material is convex with respect to the Green-Lagrange strain tensor, as anticipated.


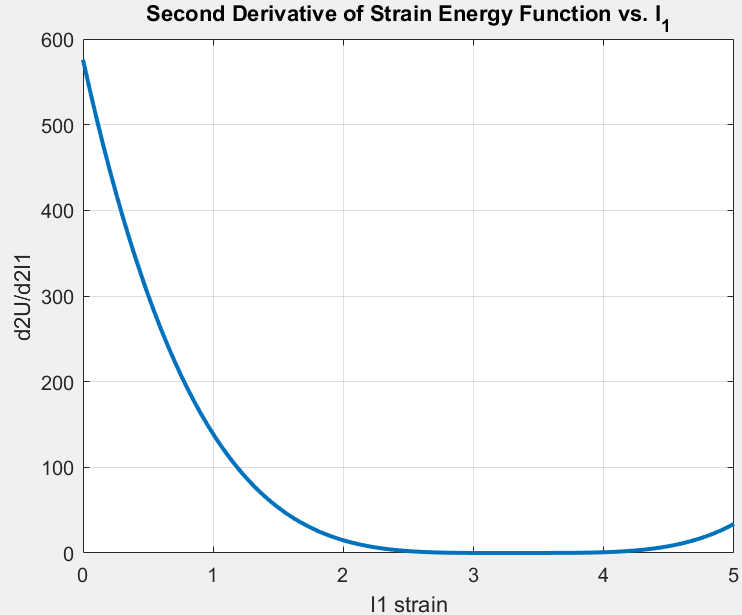

Supplement: S1 Appendix — (DOCX) [file pcbi.1012680.s001.docx]
