# Supplementary material for: Towards constructing a generalized structural 3D breathing human lung model based on experimental volumes, pressures, and strains
Source: PLoS Comput Biol. 2025 Jan 13;21(1):e1012680. doi: 10.1371/journal.pcbi.1012680 (PMC11729960; doi:10.1371/journal.pcbi.1012680)
Supplement: S2 Appendix — (DOCX) [file pcbi.1012680.s002.docx]

**Poroelastic Formulation: Permeability and Forchheimer's law**

We used a hyperfoam law coupled with a constant permeability $k$ in a poroelastic formulation to represent air diffusion in the lungs, relating air flow to gradients in pressure [1].

We assumed a fully saturated medium. Thus, Forchheimer’s law is used in ABAQUS and is expressed as follows:

$$n\times v_{w}=-\frac{k}{\gamma_{w}}\left( \frac{\partial u_{w}}{\partial x}-\rho_{w}\bar{g} \right)$$

where $n=\frac{V^{air}}{V^{total}}$ is the porosity of the lungs, which is the ratio of the volume of air (as the medium is saturated) and the total volume. The parameter $\gamma_{w}$ is the specific weight of the wetting liquid and is linked with the density of the fluid $\rho_{w}$ such that $\rho_{w}=\frac{\gamma_{w}}{g}$ where $g$ is the magnitude of the gravitational acceleration and differs from the gravitational acceleration $\bar{g}$. The quantity $u_{w}$ is the wetting liquid pore pressure at a position $x$.

We can also relate the porosity to the ratio $J$, which represents the medium’s volume in the current configuration compared to its volume in the reference configuration, such that:

$$n=1-J^{-1}\left( 1-n^{0} \right)$$

where $n^{0}$ is the initial porosity.

In Abaqus, the void ratio $e$ is generally specified and can be related to the porosity using the following equation:

$$e=\frac{n}{1-n}$$

In our case, we assigned an initial void ratio of 2, which resulted in a porosity of approximately 66%.

References:

1. Coupled pore fluid diffusion and stress analysis. Abaqus User's Manual, 2023. Link: https://classes.engineering.wustl.edu/2009/spring/mase5513/abaqus/docs/v6.6/books/usb/default.htm?startat=pt03ch06s07.html
