# Supplementary material for: Towards constructing a generalized structural 3D breathing human lung model based on experimental volumes, pressures, and strains
Source: PLoS Comput Biol. 2025 Jan 13;21(1):e1012680. doi: 10.1371/journal.pcbi.1012680 (PMC11729960; doi:10.1371/journal.pcbi.1012680)
Supplement: S3 Appendix — (DOCX) [file pcbi.1012680.s003.docx]

**Sensitivity Analysis of the Human Lung Model**

We performed a sensitivity analysis on the parameters of the human lung model. The 10 parameters were analyzed using direct sensitivity analysis. We began with a set of parameters (reference values in Table 1) and depending on the sensitivity of the parameter, we divided or multiplied the value by either 2 or 10 to define the low and high values.

*Table 1. Reference, low, and high values used to perform the sensitivity analysis.*

| **Parameters** | $\boldsymbol{\mu}$ **(kPa)** | $\boldsymbol{\alpha(-)}$ | $\boldsymbol{\nu(-)}$ | $\boldsymbol{k}_{\boldsymbol{UL,LL,UR,MR,LR}}$ **(**$\boldsymbol{10}^{\boldsymbol{-}\boldsymbol{3}}\boldsymbol{)}$**(mm^2^.s^-1^)** | $\boldsymbol{C}_{\boldsymbol{10}}$ **(kPa)** | $\boldsymbol{\lambda(-)}$ |
| --- | --- | --- | --- | --- | --- | --- |
| Reference values | $5.0$ | $1.0$ | 0.1 | $[10;10;10;10;10]$ | $1.0$ | ${1.0\text{e}}^{-2}$ |
| Low values | $2.5$ | $0.1$ | 0.05 | $[1.0;1.0;1.0;1.0;1.0]$ | $0.1$ | ${1.0\text{e}}^{-3}$ |
| High values | $10$ | $10$ | 0.2 | $[100;100;100;100;100]$ | $10$ | ${1.0\text{e}}^{-1}$ |

Following this step, we modified each value from the reference to the lower and higher ranges to study its affect on the volume, average strains, and standard deviation of the strains using the following error metric, averaged over all the points:

$$Error \left( \% \right)=100\frac{\left| R_{exp}-R_{num} \right|}{R_{exp}}$$

where $R_{num}$ and $R_{exp}$ represent the numerical and experimental results (volumes, strains), respectively.

The results are shown in the figures listing the effect on volume (Figure 1) and strain magnitudes (Figure 2) and distributions (Figure 3) below. We can see that the parameters related to the parenchyma (*µ*, *α* and *ν*) have a significant impact on the volumes and strains. At low values, the parenchyma is soft and can expand freely, compared to higher values where parameters like *µ* or *ν* “lock” the model and prevent lung expansion. The permeabilities, as expected, either limit or enhance lung expansion, depending on whether they are reduced or increased. The values slightly differ between the lobes due to the varying sizes of the lobes. The impact of permeabilities at low or high values is similar, except for the strains. When the permeabilities are low, inflation tends to be concentrated in certain regions, leading to higher average strains. However, higher permeability values allow for more complex strain distribution patterns. The stiffness of the airways contributes less at low values, as the reference value is already low. For stiffer airways (with a higher $C_{10}$), it impacts the average strains and volumes as it prevents excessive expansion. Finally, the parameter λ represents the behavior of the pleura. It is a crucial parameter that can limit the strains at higher values. Due to the bilinearity of the stress-strain curve influenced by λ, low strains are not significantly affected, but strains above 40% can be considerably limited by λ.


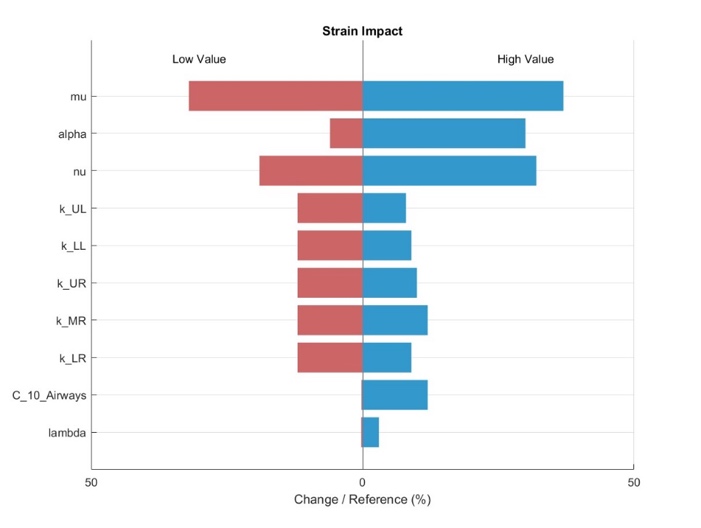

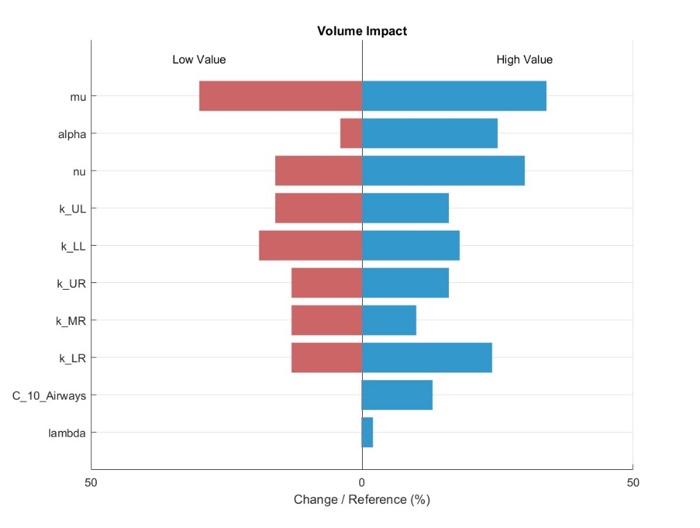
This preliminary analysis provides a fundamental interpretation of the parameters involved in this human lung model. The interactions and interconnections between the parameters are, of course, much more complex, and further sensitivity analysis may be necessary to capture all the complexities of these parameters

Figure 1. Influence of 10 parameters on lung volume

Figure 2. Influence of 10 parameters on lung strain magnitudes


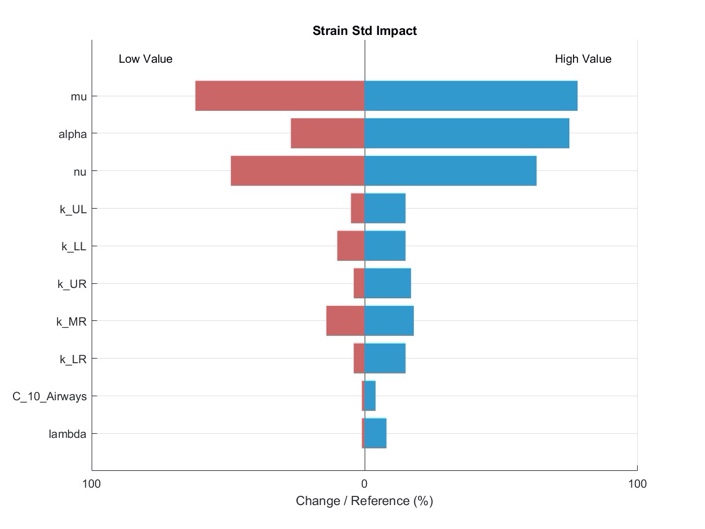


Figure 3. Influence of 10 parameters on distribution of lung strains
